# Evaluation of the content validity of patient-reported outcome (PRO) instruments developed for use with individuals with phakic presbyopia, including the Near Activity Visual Questionnaire-presbyopia (NAVQ-P) and the near vision correction independence (NVCI) instrument

**DOI:** 10.1186/s41687-021-00379-x

**Published:** 2021-10-23

**Authors:** Sarah Bentley, Amy Findley, Sima Chiva-Razavi, Christel Naujoks, Francesco Patalano, Chloe Johnson, Rob Arbuckle, James S. Wolffsohn

**Affiliations:** 1 Adelphi Values, Cheshire, UK; 2 Novartis Pharma AG, Basel, Switzerland; 3 Health and Life Sciences, Aston University, Birmingham, UK

**Keywords:** Presbyopia, Content validity, Patient-reported outcome, Cognitive debriefing, Qualitative, Interviews, NAVQ-P

## Abstract

**Background:**

Presbyopia is the age-related deterioration in the ability to focus on close objects. In order to develop a patient-reported outcome (PRO) instrument to assess near vision functioning, the Near Activity Visual Questionnaire (NAVQ) was adapted to incorporate modern technology (e.g. smartphones) and to be appropriate for use in phakic presbyopia, leading to the development of the NAVQ-Presbyopia (NAVQ-P). Additional single-item instruments of near vision correction independence (NVCI), correction preference (NVCP), and vision satisfaction (NVS) were also developed. The study aimed to evaluate the content validity of the NAVQ-P and additional instruments in individuals with phakic presbyopia.

**Methods:**

Participants in the US (n = 15), Germany (n = 10) and France (n = 10) took part in face-to-face, qualitative, cognitive debriefing interviews. Seven healthcare professionals (HCPs) were also interviewed to assess the clinical relevance of the PRO instruments. Interviews started with open-ended qualitative concept elicitation questioning; participants then completed the PRO instruments on an electronic tablet using a “think-aloud” process and were asked about their understanding and relevance of each item, instruction, response scale and recall period. Interviews were conducted in two rounds allowing for modifications between rounds.

**Results:**

The participants interpreted the majority of the PRO instruments and recall period correctly and consistently. They were able to select an appropriate response option without difficulty. Minor modifications were made to the PRO instruments based on interview findings. Instruction/item wording was modified to include reference to use of a magnifying glass, in addition to glasses and contact lenses. Two items were added to assess difficulty with precision tasks (e.g. sewing) and taking longer to adjust from distance to near vision. HCPs confirmed the relevance of the concepts being measured for presbyopia and recommended the addition of an item assessing contrast sensitivity.

**Conclusions:**

Developed in accordance with the FDA PRO Guidance, the findings support content validity of the NAVQ-P as a suitable, well-understood instrument of relevant near vision functioning concepts in individuals with phakic presbyopia. The NVCI and additional PRO instruments are appropriate to assess near vision correction independence, correction preference, and vision satisfaction. Future work will assess the psychometric properties of the NAVQ-P and additional PRO instruments.

**Supplementary Information:**

The online version contains supplementary material available at 10.1186/s41687-021-00379-x.

## Introduction

Presbyopia occurs when the physiologically normal age-related reduction in the eye's focusing range reaches a point, when optimally corrected for distance vision, that the clarity of vision at near is insufficient to satisfy an individual's requirements [1, 2]. It is hypothesized to be caused by a loss of lens elasticity preventing focal point change [3, 4]. While the etiology of this condition is not fully elucidated, recent research suggests that an increase in lens rigidity is the primary causative mechanism [5, 6]. Presbyopia is expected to be experienced in about 80% of people aged 40 years or above [1]. Individuals with presbyopia have difficulty with near vision function tasks (e.g. reading or threading a needle [7, 8]) and experience burden associated with wearing glasses [9]. Consequently, presbyopia has a significant impact on individuals’ health related quality of life [7, 8, 10–14] and entails substantial humanistic and economic burden [15].

Existing clinical assessment tools (such as visual acuity assessment through use of a standardised Snellen chart) lack adequate assessment of the individual experience of presbyopia, highlighting the need for patient-reported outcome (PRO) instruments in this specific population. A recent literature review found there was a paucity of PROs developed for use in phakic presbyopia in line with the Food and Drug Administration (FDA) PRO guidance [16]. Phakic presbyopia is presbyopia that occurs for an individual who still has a natural lens, as opposed to pseudophakic presbyopia where the individual no longer has a natural lens (such as following surgery). There are a number of treatment considerations when managing pseudophakic presbyopia in comparison to phakic presbyopia such as navigating corneal scars and residual corneal irregularities from prior incisions.{Paley [17]} Instruments such as the Near Activity Visual Questionnaire (NAVQ) [18], National Eye Institute Visual Function Questionnaire (NEI VFQ-25) [19], National Eye Institute Refractive Error Quality of Life Instrument-42 (NEI RQL-42) [20] were among those identified as PRO instruments that assess vision outcomes of presbyopia or similar conditions. The FDA guidance outlines the requirement of evidence of content validity in a given context of use for existing, modified, or newly created PRO instruments used to support claims in approved medical product labelling [16, 21]. The NAVQ was the only PRO identified by the literature review as a suitable instrument to assess patient-reported near vision function [16, 18]. The other identified instruments had limitations such as lack of focus on presbyopia and insufficient evidence to support the psychometric properties.

The NAVQ was originally developed and validated in a population which included individuals with pseudophakic presbyopia [18]. Although the NAVQ was developed in line with the FDA guidance, modifications were required to ensure the NAVQ was suitable for use in clinical trials with a purely phakic population, and that the instrument assessed difficulties experienced with the use of modern information technology devices due to near-vision problems (smartphones, computers, and tablet devices which were not as widely used when the NAVQ was first developed). To inform modifications to the NAVQ, first an update of the literature review and a social media listening study was conducted to explore the lived experience of presbyopia [16, 22]. This information was used to generate a preliminary conceptual model to summarize the key symptoms and impacts in presbyopia.

Based on the preliminary conceptual model, the NAVQ was adapted for use with individuals with phakic presbyopia and the resulting instrument is called the NAVQ-Presbyopia (NAVQ-P). Additional single-item instruments for the assessment of near vision correction independence (NVCI), near vision correction preference (NVCP) and near vision satisfaction (NVS) were also developed, along with two global items to assess patient global impression of severity of near vision function (PGIS-Presbyopia) and patient global impression of change in near vision function (PGIC-Presbyopia). All instruments were designed for electronic completion using a tablet device. Cognitive debriefing (CD) of the electronic NAVQ-P, NVCI and additional instruments was required to evaluate content validity of the instruments in individuals with phakic presbyopia.

The aim of the study was to evaluate the content validity of the NAVQ-P, NVCI and the additional instruments (NVCI, NVCP, NVS, PGIS-Presbyopia and PGIC-Presbyopia) in individuals with phakic presbyopia through the conduct of qualitative research, with the ultimate objective to develop PRO instruments suitable for use as clinical trial endpoints in phakic presbyopia.

## Methods

### Study design

This was a non-interventional, qualitative study involving participants with phakic presbyopia and healthcare professionals (HCPs). In-depth, face-to-face, semi-structured interviews were conducted with thirty-five individuals with phakic presbyopia to assess the content validity of the PRO instruments. Seven additional interviews were conducted with HCPs to obtain a clinical perspective on the relevance of the items included within the PRO instruments.

**Fig. 1:**
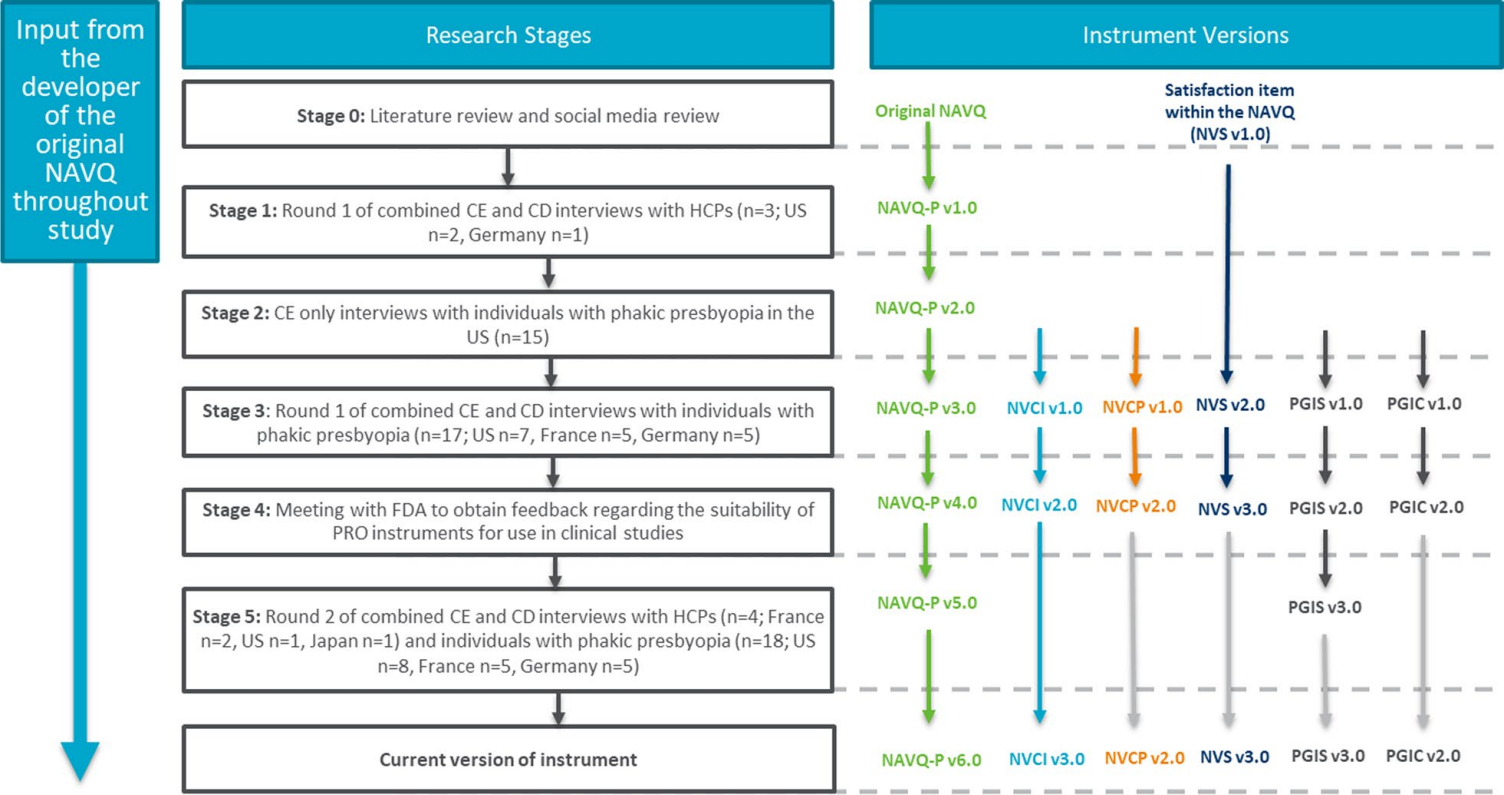
Study methodology. *CE concept elicitation, *CD* cognitive debriefing, *HCP* healthcare professional, *FDA* US Food and Drug Administration, *PRO* patient-reported outcome, *NAVQ-P* Near Activity Visual Questionnaire for Presbyopia, *NVCI* Near Vision Correction Independence, *NVCP* Near Vision Correction Preference, *NVS* Near Vision Satisfaction, *PGIS* Patient Global Impression of Severity, *PGIC* Patient Global Impression of Change

There were five stages to the research as outlined in Fig. 1. A purposive sampling approach involved enrolment of individuals with phakic presbyopia in the US (n = 15), France (n = 10) and Germany (n = 10), and HCP representation from the US (n = 3), France (n = 2), Japan (n = 1), and Germany (n = 1). These countries were selected to provide a representation from the US and Europe, and a HCP from Japan was interviewed to explore if there were any apparent differences in Asia. Both samples participated in combined concept elicitation (CE) and CD interviews. Findings from the CE section of the interviews (stage 2) have been published separately and are therefore not described in this article [23].

CD interviews were conducted across two rounds with individuals with phakic presbyopia (round 1/stage 3: n = 17; round 2/stage 5: n = 18) and HCPs (round 1/stage 1: n = 3; round 2/stage 5: n = 4). Conducting the interviews in rounds enabled iterative modifications to the NAVQ-P, NVCI and additional instruments to be tested in a subsequent round of interviews (Fig. 1). Expert clinical input to study design, findings and instrument modification was provided by the developer of the original NAVQ [18]. Ethical approval was obtained in accordance with requirements per study country (Additional file 1).

### Study sample

Partner recruitment agencies worked with ophthalmologists/optometrists to recruit individuals with phakic presbyopia who met the inclusion and exclusion criteria (Additional file 1). Referring ophthalmologists/optometrists confirmed the participant’s eligibility by completing a Case Report Form (CRF) and ensured written informed consent was obtained using an Information and Consent Form (ICF) prior to any other study activities and prior to any personal data being shared (Additional file 1). The recruitment agencies collected demographic information using the demographic form and participants were remunerated for taking part. HCPs (N = 7) were identified based on their area of expertise and contribution to the field. HCPs (ophthalmologists or optometrists) were recruited from the US, Germany, France, and Japan.

### Cognitive debriefing interview procedure

The aim of the CD interviews with individuals with phakic presbyopia was to assess relevance and understanding of item wording, instructions, recall period and response options of the PRO instruments and the usability of the electronic PRO (ePRO) tablet device (Samsung Galaxy Tab E). Given seeing small text on a digital screen was identified in the previous literature as an impact of presbyopia, the ePRO was developed with this in mind (Roboto font size 23) and participants were asked specifically about whether they found the font size easy to read. Interview administration was not considered given the design of the ePRO was developed to be easy for individuals with phakic presbyopia to read.

The interviews were conducted by trained, experienced interviewers using a semi-structured interview guide (Additional files 2 and 3). Minor updates were made to the interview guide between rounds of interviews to correspond with modifications made to the PRO instruments. Additional probes were also added to the interview guide following round 1 (stage 3) interviews to explore any differences between participants who had comorbid myopia and those who did not. Figure 1 outlines the instrument version debriefed at each stage of the study. The study team sought feedback from the US Food and Drug Administration (FDA) between round 1 and round 2 interviews (stage 4).

A ‘think-aloud’ process was employed which involved participants being asked to speak their thoughts aloud as they read all instructions and completed each item on the ePRO device [21]. Specifically, interviews with individuals with phakic presbyopia utilised ‘think aloud’ discussion to elicit in-depth evidence on the understanding, relevance and interpretation of the PRO instruments. Targeted probing was used to ensure elicitation of feedback on item relevance and whether any important concepts were missing. The interview process was designed in line with best practice and regulatory standards in the assessment of content validity (FDA Guidance, International Society for Pharmacoeconomics and Outcomes Research (ISPOR) Good Research Practice [21, 24–26]). All interviews with individuals with phakic presbyopia were conducted in the participant’s local language and lasted approximately 60 min (approximately 45 min was spent on the CD part of the interview).

HCPs were asked to provide feedback on the item wording, whether they perceived the concept to be relevant to phakic presbyopia, missing concepts, and any comments on the response options or recall period. All HCP interviews were conducted by telephone in English and lasted approximately 60 min (approximately 30 min was spent on the CD part of the interview).

### Data analysis

Planned analyses and subgroup analyses was detailed in a qualitative analysis plan (QAP) prior to data collection. Verbatim transcripts were qualitatively analyzed using thematic analysis methods and ATLAS.ti software [27, 28]. Verbatim patient quotes were highlighted and grouped by theme/topic. Frequency counts were generated per item and instruction of the NAVQ-P, NVCI and additional PRO instruments to indicate understanding and relevance (yes/no/unclear), along with the generation of a list of participant verbatim statements for each coding domain [29]. Subgroup comparisons identified patterns in instrument interpretation between individuals with phakic presbyopia according to presbyopia severity (mild vs. moderate/severe), age of participant, country (US vs. France vs. Germany), and presence of co-morbid myopia or not, in line with the sampling quotas specified (Additional file 1).

## Results

### Demographic and clinical characteristics

#### *HCPs (N* = *7)*

HCPs were interviewed in the US (n = 3/7, 42.9%), France (n = 2/7, 28.6%), Japan (n = 1/7, 14.3%) and Germany (n = 1/7, 14.3%). HCPs were practicing ophthalmologists (n = 5/7, 71.4%) or optometrists (n = 2/7, 28.6%). All HCPs had spent over 10 years managing individuals with presbyopia and self-reported treating an average of > 31 individuals with presbyopia every month (n = 5/7, 71.4%) or 21–30 individuals per month (n = 2/7, 28.6%). HCPs reported that their routine appointments with individuals with presbyopia were typically approximately once per year (n = 3/7, 42.9%), twice per year (n = 2/7, 28.6%) or monthly (n = 2/7, 28.6%). Four HCPs (n = 4/7, 57.1%) reported that they experienced presbyopia themselves, two HCPs (n = 2/7, 28.6%) reported that they did not, and one HCP (n = 1/7, 14.3%) did not comment on whether they experienced presbyopia themselves or not. Table 1 provides an overview of HCP demographic characteristics.

**Table 1:** Healthcare professional demographic information

Description	Total (N = 7)
Age	
Average (range)	52.1 (41–69)
Gender, n (%)	
Male	6 (85.7)
Female	1 (14.3)
Role, n (%)	
Ophthalmologist	5 (71.4)
Optometrist	2 (28.6)
Years in current position, n (%)	
Less than 1 year	1 (14.3)
1–5 years	1 (14.3)
5–10 years	1 (14.3)
Longer than 10 years	4 (57.1)
Time spent managing individuals with presbyopia, n (%)	
10 + years	7 (100)
Average number of individuals with presbyopia seen each month, n (%)	
> 31 individuals	5 (71.4)
21–30 individuals	2 (28.6)
Would you typically diagnose individuals with presbyopia? n (%)	
Yes	7 (100)
No	0
If yes which methods do you use? n (%)*	
Refraction	7 (100)
Visual acuity	6 (85.7)
Slit lamp	2 (28.6)
Examination of retina	2 (28.6)
How often do you see individuals with presbyopia for routine appointments? n (%)	
Once per year	3 (42.9)
Twice per year	2 (28.6)
Monthly	2 (28.6)
Do you experience presbyopia yourself? n (%)	
Yes	4 (57.1)
No	2 (28.6)
Missing data	1 (14.3)

*Multiple answers possible

#### Individuals with phakic presbyopia (N = 35)

The thirty-five individuals with phakic presbyopia interviewed were based across the US (n = 15), France (n = 10) and Germany (n = 10). The mean age of the sample was 53.5 years old (range: 40–65). There were more females (n = 21/35, 60.0%) than males (n = 14/35, 40.0%) interviewed. A similar number of Caucasian (n = 11/35, 36.7%) and Black/African American (n = 9/35, 30.0%) participants were interviewed. Data on race or ethnicity was not obtained for the French participants (n = 10/35, 28.6%) in line with French legislation.

There were a higher percentage of participants who had ‘moderate-severe’ phakic presbyopia (n = 21/35, 60.0%) compared with ‘mild’ phakic presbyopia (n = 14/35, 40.0%); based on their near addition (ADD) results provided by the referring physician. Most participants (n = 22/35, 62.9%) were using glasses for near vision correction except for six participants who used contact lenses (n = 6/35, 17.1%). Data on current correction method was missing for eight participants (n = 8/35, 22.9%). Table 2 provides an overview of participant demographic and clinical characteristics.

**Table 2:** Demographic and clinical characteristics of individuals with phakic presbyopia

Description	France (N = 10)	Germany (N = 10)	USA (N = 15)	Total (N = 35)
*Participant demographic characteristics*				
Age				
Average (range)	55.9 (41–65)	51.1 (40–63)	53.6 (41–65)	53.5 (40–65)
Sex, n (%)				
Male	2 (20.0)	5 (50.0)	7 (46.7)	14 (40.0)
Female	8 (80.0)	5 (50.0)	8 (53.3)	21 (60.0)
Race, n (%)				
Caucasian	Not appropriate to collect in France	7 (70.0)	4 (26.7)	11 (36.7)
Black/African American		1 (10.0)	8 (53.3)	9 (30.0)
Asian		2 (20.0)	–	2 (6.7)
Other—Hispanic		–	3 (20.0)	3 (8.6)
Missing data	10 (100)	–	–	10 (28.6)
Participant self-reported severity of presbyopia, n (%)				
Very severe	1 (10.0)	1 (10.0)	1 (6.7)	3 (8.6)
Severe	1 (10.0)	3 (30.0)	4 (26.7)	8 (22.9)
Moderate	7 (70.0)	–	7 (46.7)	14 (40.0)
Mild	1 (10.0)	6 (60.0)	3 (20.0)	10 (28.6)
Participant self-reported myopia*				
No	5 (50.0)	6 (60.0)	8 (53.3)	19 (54.3)
Yes	5 (50.0)	4 (40.0)	7 (46.7)	16 (45.7)
*Participant clinical characteristics (reported by recruiting clinician)*				
Number of years since diagnosis*				
Average (range)	10.4 (0.5–20.9)	7.3 (1–17.1)	9.9 (0.2–34.6)	9.3 (0.2–34.6)
Visual acuity score, average (range)^a^				
Left eye (decimal)	0.92 (0.6–1.0)	0.67 (0.5–0.8)	0.55 (0.4–0.7)	0.68 (0.4–1.0)
Right eye (decimal)	0.92 (0.6–1.0)	0.64 (0.4–0.8)	0.55 (0.3–1.0)	0.68 (0.3–1.0)
Severity of participants’ binocular DCNVA at 40 cm, n (%)				
Mild	2 (20.0)	6 (60.0)	5 (33.3)	13 (37.1)
Moderate-severe	3 (30.0)	4 (40.0)	10 (66.7)	17 (48.6)
Information not available	5 (50.0)	–	–	5 (14.3)
Severity of participants’ near ADD, n (%)				
Mild	3 (30.0)	6 (60.0)	5 (33.3)	14 (40.0)
Moderate-severe	7 (70.0)	4 (40.0)	10 (66.7)	21 (60.0)
Clinician reported myopia/ near sightedness †				
None	6 (60.0)	5 (50.0)	8 (53.3)	19 (54.3)
Mild	2 (20.0)	–	2 (13.3)	4 (11.4)
Moderate	1 (10.0)	–	2 (13.3)	3 (8.6)
High	1 (10.0)	–	3 (20.0)	4 (11.4)
Missing data	–	5 (50.0)	–	5 (14.3)
Concomitant conditions, n (%)^				
Yes:	1 (10.0)	–	2 (13.3)	3 (8.6)
Posterior detachment of the left vitreous	1 (10.0)	–	–	1 (2.9)
Asthma	–	–	1 (6.7)	1 (2.9)
Glaucoma	–	–	1 (6.7)	1 (2.9)
Chronic obstructive pulmonary disease	–	–	1 (6.7)	1 (2.9)
Current treatment for presbyopia, n (%)^				
Glasses	7 (70.0)	5 (50.0)	10 (66.7)	22 (62.9)
Unspecified	1/7 (14.3)	5/5 (100)	8/10 (80.0)	14 (40.0)
Single vision	2/7 (28.6)	–	2/10 (20.0)	4 (11.4)
Multifocal	4/7 (57.1)	–	–	4 (11.4)
Contact lenses	–	5 (50.0)	1 (6.7)	6 (17.1)
Missing data	3 (30.0)	–	5 (33.3)	8 (22.9)

*One participant’s data was removed in this category only as it appeared to have an error

^a^Two participants data missing. ^Multiple answers possible. ~ This question was not collected on the demographic form in Germany so this information has been generated from what the patient reported during the interview. In addition, one patient reported myopia in the demographic form but reported that they did not have myopia during the interview. The clinician also reported that this patient did not have myopia, so this patient has not been counted as a myopic patient in the analyses of findings. †Clinicians were not asked to confirm diagnosis of myopia for the round 1 interviews

### Stage 1: HCP interviews (round 1, n = 3) CD findings

Three HCPs (US n = 2, Germany n = 1) were debriefed the NAVQ-P v1.0 and NVS v1.0 (at this point the satisfaction question formed part of the NAVQ). These HCPs indicated that the NAVQ-P assessed concepts relevant to individuals with phakic presbyopia. Two HCPs (n = 2/3, 66.7%) noted that the instructions in the NAVQ-P v1.0 may cause individuals who have co-morbid myopia to answer incorrectly, given that they take their glasses off to be able to see up close. As a result, the instructions were updated from ‘… when you were not wearing glasses/contact lenses’, to ‘… when you were not wearing glasses/contact lenses to see things close to you (less than an arm’s length away)’.

“But people that are nearsighted who don’t have anything on, no glasses, no contact lenses, nothing, if they’re nearsighted to the proper degree, they will never have any symptomatic presbyopia as long as their glasses and their contact lenses are not used.” (HCP 2)

Minor changes were recommended to two items to ensure that the visual task examples provided in items were of equivalent difficulty. This included removal of the example ‘items on a menu’ from item 1 (which assessed reading small print) since this task may not be equivalent to other provided examples (e.g. newspaper), given the potential for dimmed lighting in restaurant settings. The example of ‘gardening’ was also removed from item 9 (which assessed seeing objects up close to engage in hobbies) as the HCPs felt that gardening mostly involved intermediate vision. Additionally, an item to assess contrast sensitivity was added to the NAVQ-P v1.0 based on HCP feedback. All revisions were implemented ahead of the intermediary participant CE interviews (NAVQ-P v2.0).

“Not necessarily because newspaper people might have more light than perhaps in a dimly lit restaurant.” (HCP1)

“The number of tasks that you do that are actually near tasks are pretty small. I mean you’re digging a hole and you’re raking the, the ground and you’re, you know—gardening is not a, a near vision intense hobby.” (HCP2)

### Stage 2: Interviews with individuals with phakic presbyopia (CE-only, n = 15) CE findings

Findings from an initial round of CE interviews with a separate sample of 15 individuals with phakic presbyopia (independent of combined CE and CD rounds) contributed to the modification of the NAVQ-P v2.0 forming the NAVQ-P v3.0. Revisions included the addition of relevant item examples, inclusion of an item to assess seeing objects up close in bright light, and the rewording of an item which assessed difficulties with contrast sensitivity to ensure that language was patient-friendly. The item which assessed satisfaction with near vision (from the original NAVQ) was separated from the NAVQ-P v2.0 to form the Near Vision Satisfaction (NVS) instrument. Four other single-item instruments were also created: NVCI, NVCP, PGIS-Presbyopia, and PGIC-Presbyopia.

### Stage 3: Interviews with individuals with phakic presbyopia (round 1, n = 17) CD findings

**Fig. 2:**
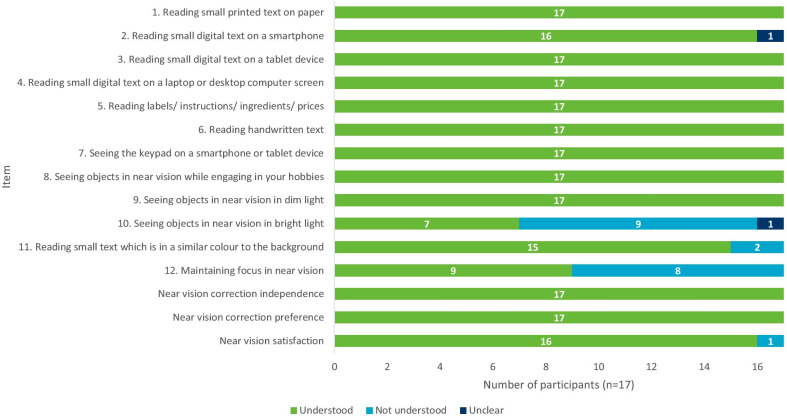
Summary of item understanding during round 1 CD interviews

**Fig. 3:**
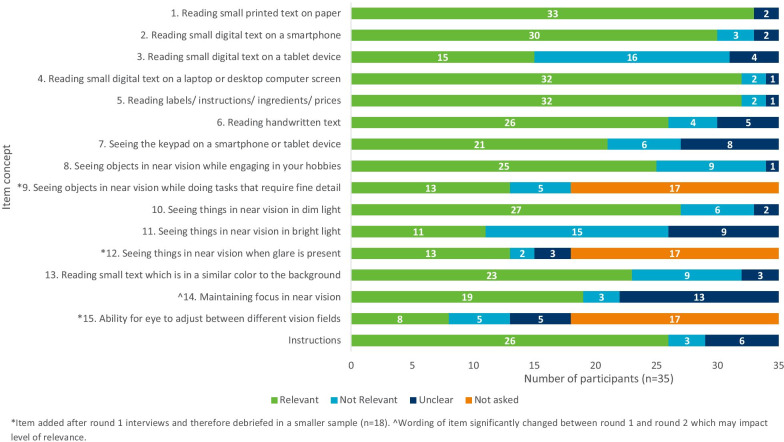
Summary of overall conceptual relevance (round 1 and round 2 combined)

Item wording, response options and the recall period of the NAVQ-P v3.0 and additional instruments were generally well understood and participants appeared to interpret most items correctly and consistently (Fig. 2). Three NAVQ-P v3.0 items were misunderstood in round 1 interviews (summarized in Table 3). These items were misunderstood by two or more participants including item 1 (which assessed seeing objects in bright light, understood by n = 7/17, 41.2%), items 11 (which assessed contrast sensitivity, understood by n = 15/17, 58.8%), and item 12 (which assessed maintaining focus for near vision activities, understood by n = 8/17, 52.9%). Overall conceptual relevance of the NAVQ-P v3.0 was analyzed collectively across the two rounds of CD interviews (Fig. 3).

“That needs to be changed …it says seeing objects close to you in bright light such as seeing dashboard in a car. The dashboard doesn’t have lighting that bright …What you see is the light that reflects out there that comes.” (F55-MILD-R1-US3)

**Table 3:** Modifications made following round 1 and round 2 CD interviews

Item	Understanding	Description	Modifications
Round 1			
Item assessing seeing objects up close while engaging in hobbies	n = 17/17 (100%)	Ten patients (n = 10/17, 58.8%) discussed the examples provided of hobbies requiring near vision (e.g. playing card games, sewing, seeing photographs). Of these patients, the majority (n = 7/10, 70.0%) felt that the examples varied in difficulty. Specifically, all seven patients felt that sewing was more challenging than the other examples provided “Well sewing is much more difficult. That would be extremely difficult. Um, card games would be pretty easy and photographs would be okay. Um, yeah. Those, those are fine.” (M65-MOD-R1-US6)	Examples were revised from ‘playing card games, sewing, seeing photographs’ to ‘playing board games, completing puzzles, arts and crafts activities’
Item assessing seeing things in dim light	n = 17/17 (100%)	Ten patients (n = 10/17, 58.8%) suggested additional instances where they had experienced difficulties seeing objects in dim or poor light. Examples included: reading a book at night (n = 5/10, 50.0%), seeing objects when in a bar or restaurant (n = 2/10, 20.0%), entering a combination on a safe (n = 1/10, 10.0%; F54-MILD-R1-US5), reading instruction manuals (n = 1/10; 10.0%; M61-MOD-R1-DE2) and looking at maps in poor lighting (n = 1/10, 10.0%; M53-MILD-R1-DE3) “Well, for me … because as I was telling you, I thought about restaurants, bars, etc., whereas it can simply relate to a small bedside lamp when you read, and you can have difficulty in this case.” (F41-MILD-R1-FR4)	Example of ‘reading a book by lamp light’ was added
Item assessing difficulty with near vision in bright light	n = 7/17 (41.2%)	Ten participants focused on ‘glare’ rather than their vision in bright light “That needs to be changed …it says seeing objects close to you in bright light such as seeing dashboard in a car. The dashboard doesn’t have lighting that bright …What you see is the light that reflects out there that comes.” (F55-MILD-R1-US3)	Examples revised from ‘seeing the dashboard in a car’ to ‘reading outdoors in daylight, seeing details on objects in a brightly lit store’
Item assessing difficulty seeing contrasts in near vision	n = 15/17 (88.2%)	Two participants did not understand the description of contrast and instead answered the question based on reading small text only “How much difficulty have you had reading small text which is in a similar color to the background, such as website and leaflets? Never thought about that. Small text, it's getting a moderate difficulty.” (F54-MILD-R1-US5)	No modifications made
Item assessing difficulty maintaining focus in near vision	n = 9/17 (52.9%)	Seven participants described mental focus rather than visual focus “…yes, but it’s much more difficult to… I’d say, to explain. It’s essentially impressions. So, well … tell you that I have precisely difficulties remaining focused… I can’t… I can’t assure you of it.” (M51-MOD-R1-FR1)	Item 12 was reworded
New item added	N/A	Thirteen patients (n = 13/32, 40.6%) reported having difficulty completing precision tasks that required them to see small details in their near vision during the CE interviews and round 1 of the combined CE/CD interviews. Of these patients, nine (n = 9/13, 69.2%) described this experience spontaneously during their interview. The most common type of precision activities reported by patients was sewing (n = 10)	Item to assess seeing fine detail was developed
New item added	N/A	Nine patients (n = 9/32, 28.1%) during the CE interviews and round 1 of the combined CE/CD interviews described their eyes taking longer time to adjust to see things when the distance of vision changes, as a result of presbyopia. Eight of these nine patients (88.9%) reported this concept spontaneously	Item to assess difficulty adjusting from far vision to close vision was developed
Near vision correction independence item	n = 17/17 (100%)	The majority of participants (n = 14/17, 82.4%) were able to select an appropriate response to this item without difficulty Three patients (n = 3/17, 17.6%) provided more detailed feedback on the response options. One patient (n = 1/3, 5.9%; F58-MOD-R1-FR1) indicated that she would ideally want to respond ‘3/4 of the time’, which is not captured in the current response options. The other two patients (n = 2/3, 66.7%) felt the response options were too broad. FDA also provided feedback on the response options “That's a bit funny now, because of all the activities. In the last 7 days. Less than half. That's very wide. Above all: At night you usually don't wear glasses. Perhaps it would be better to wear them during the day. That's my opinion.” (M53-MILD-R1-DE3)	Updated to include ‘magnifying glass’ as a method of vision correction. An alternative set of response options (‘Never’ to ‘Always’) was developed for the NVCI to be debriefed alongside the original response options (‘None of the time’ to ‘All of the time’) in round 2
Near vision satisfaction item	n = 16/17 (94.1%)	Minor wording changes were implemented ahead of the next round of interviews to ensure that the patients were considering their vision without using any vision correction aids (e.g. glasses, contact lenses or a magnifying glass)	Updated to include ‘magnifying glass’ as a method of vision correction
Near vision correction preference item	n = 17/17 (100%)	The project team decided that this item would be more useful if the response options described the actual correction methods that would be expected to be used	The response options in the NVCP were updated to describe actual correction methods, rather than the options of ‘study treatment’ and ‘treatment before study’
Patient global impression of severity of presbyopia item	n = 17/17 (100%)	The PGIS-Presbyopia item was updated to align with FDA-recommended example items (outlined in Patient Focused Drug Development Guidance (Guidance 3)) [30]	The PGIS-Presbyopia response options were updated to remove ‘Very mild’ and include ‘Very severe’
Patient global impression of change in presbyopia item	n = 16/17 (94.1%)	The PGIC-Presbyopia item was updated to align with FDA-recommended example items (outlined in Patient Focused Drug Development Guidance (Guidance 3)) [30]	The PGIC-Presbyopia response options were updated to reduce the number of options from seven to five: ‘Much worse’, ‘A little worse’, ‘No change’, ‘A little better’, ‘Much better’
Round 2			
Item assessing difficulty in reading small printed text on paper	n = 16/18 (88.9%)	Misinterpreted by two participants to be asking about reading from a tablet device (n = 1) or a smartphone (n = 1) “Because sometimes you have the tablet and want to read something and wonder if you see this correctly.” (M47-MILD-R2-DE2)	Item formatting updated to bolden the words ‘on paper’
Item assessing difficulty reading small digital text on a tablet device	n = 15/18 (83.3%)	Misinterpreted by two participants to be asking about reading from a laptop (n = 1) or a smartphone (n = 1). Two participants (n = 2/18, 11.1%) overlooked the instruction ‘without increasing font size’ “…but to me, the tablet, it’s my smartphone” (M56-MILD-R2-FR6)	Item formatting updated to bolden the words ‘without increasing font size’
Item assessing difficulty seeing the keypad on a smartphone or tablet screen	n = 12/18 (66.7%)	Six participants incorrectly interpreted ‘keypad’ as referring to computer keyboards or buttons on their mobile phone “A keypad is something where you press something down, not a touchscreen.” (F40-MILD-R2-DE1)	Item wording was changed from ‘device’ to ‘screen’ to improve comprehension
Item assessing seeing things in dim light	n = 17/18 (94.4%)	Six participants (n = 6/18, 33.3%) suggested additional examples of activities where they had experienced difficulties seeing objects in dim or poor light. This included: reading from a TV screen (n = 3/6, 50.0%), watching movies (n = 1/6, 16.7%; F42-MOD-R2-US3), reading in bed at night (n = 1/6, 16.7%; F60-MOD-R2-US4) and reading outside at dusk (n = 1/6, 16.7%; F60-MOD-R2-US4) “Um, even at night if you’re just trying to read a book in bed and not disturbing the other person, you have your book light on, it’s difficult.” (F60-MOD-R2-US4)	The example provided was revised to better represent the lighting condition specified
Item assessing ability for the eye to adjust between different distances	n = 13/18 (72.2%)	Five participants either had a general misunderstanding of the concept (n = 3) or focussed on a change in lighting conditions due to the example provided (n = 2) INTERVIEWER: “How would you ask this question in your own words?” PATIENT: “How difficult is it for you to see things in different lighting?” (F48-MOD-R2-US1)	Item revised from referring to ‘looking out the window’ to ‘look across the room’ to avoid the risk of responses being influenced by the different lighting and weather conditions which may impact vision outdoors

#### Round 1 instrument modifications

Modifications were made to the NAVQ-P v3.0 (becoming NAVQ-P v4.0) based on Round 1 interview findings, including the addition of ‘magnifying glass’ alongside ‘wearing glasses/contact lenses’ in the instructions and item stem (the first part of each question) to ensure that individuals would consider all possible forms of vision correction. The second instruction (which read ‘If you did not do the described activity or you have stopped for reasons that are not related to your vision then please select the ‘N/A or stopped doing this for non-visual reasons' option’) was removed as it was determined to be redundant. Revisions were made to the ‘not applicable’ response option wording to read: ‘I did not do this activity in the past seven days’. Examples within three items were updated, one item was reworded and two new items to assess ‘seeing fine detail’ and ‘difficulty adjusting from far vision to close vision’ were developed. Updates were also made to the NVCI, NVCP, NVS, PGIS-Presbyopia and PGIC-Presbyopia (see Table 3 for further information about modifications made to all items). Notably, an alternative set of response options (‘Never’ to ‘Always’) was developed for the NCVI to be debriefed alongside the original response options (‘None of the time’ to ‘All of the time’) in round 2.

### Stage 4: FDA feedback on NAVQ-P v4.0 and additional instruments (Type-C meeting)

Based on FDA feedback obtained, the NAVQ-P v4.0 was updated to form v5.0. Modifications were made to the instructions, item stem, and four items. One new item was incorporated to assess the ability to see things when glare is present. Findings from round 1 qualitative interviews identified that this concept was a distinct construct to difficulty seeing things in bright light, thus, an additional item was warranted. Additional wording modifications were made to the PGIS-Presbyopia following feedback from the FDA to create PGIS-Presbyopia v3.0. No further changes were made to the NVCI v2.0, NVCP v1.0, NVS v2.0, and the PGIC-Presbyopia v2.0.

### Stage 5: HCP interviews (round 2, n = 4) CD findings

Four HCPs (France n = 2, US n = 1, Japan = 1) debriefed the instruments in round 2 (see versions in Fig. 1). These HCPs agreed that the NAVQ-P v5.0 and additional instruments would be well understood by individuals with phakic presbyopia and supported the clinical relevance of concepts assessed. No missing concepts of importance were highlighted. Minor wording changes were suggested, mainly concerning the examples provided within item wording. The changes were revisited following round 2 of CD interviews with individuals with phakic presbyopia to ensure that revisions were in-line with participant feedback and understanding.

“The only part of it that might get confusing is, um, you’re saying, um, close to you in dim light, but then you use the term reading a book by lamplight. So what you’re inherently doing in this second portion of the questions, is, um, it’s, it’s almost like the patient is- they’re not in dim light anymore because they are beside a lamplight.” (HCP4)

### Stage 5: Interviews with individuals with presbyopia (round 2, n = 18) CD findings

**Fig. 4:**
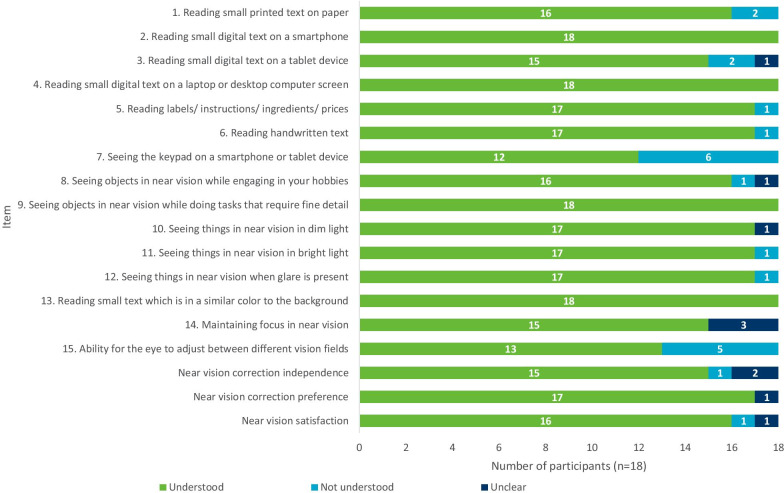
Summary of item understanding during round 2 CD interviews

Item wording, response options and recall period of the NAVQ-P v5.0 and additional instruments were generally well understood and interpreted consistently (Fig. 4).

Minor issues in understanding or consistent interpretation were identified for four NAVQ-P v5.0 items (Table 3). Item 1 (difficulty reading small printed text on paper) was misinterpreted by n = 2/18 (11.1%) as asking about reading from a tablet or smartphone. Item 3 (difficulty reading small digital text on a tablet device) was misinterpreted by n = 2/18 (11.1%) to be about reading from a laptop. Item 7 (difficulty seeing the keypad on a smartphone or tablet screen) demonstrated an inconsistent interpretation of ‘keypad’, with n = 6/18 (33.3%) participants discussing keyboards or buttons rather than focussing on the keypad on the screen. Item 15 (ability for the eye to adjust between different vision fields) was generally misunderstood by n = 3/18 (16.7%) and also misinterpreted by n = 2/18 (11.1%) to relate to a change in lighting.

“A keypad is something where you press something down, not a touchscreen.” (F40-MILD-R2-DE1)

#### Round 2 instrument modifications

Modifications to NAVQ-P v5.0 item wording and response options were made (forming NAVQ-P v6.0). See Table 3 for further information about updates to items. Item ordering was also adjusted: the order of the items assessing ‘vision when glare is present’ and ‘vision in bright light’ was reversed to avoid respondents thinking about glare when responding to the bright light item. The alternative set of response options were retained for the NVCI (v3.0). No changes were made to the NVCP v2.0, NVS 3.0, PGIS-Presbyopia v3.0 and PGIC-Presbyopia v2.0.

### Item relevance assessed across round 1 and round 2 interviews with individuals with phakic presbyopia (n = 35)

Given the concepts assessed did not change across both rounds (apart from some new items being added), relevance is summarised collectively for both round 1 (stage 3) and round 2 (stage 5) interviews with individuals with phakic presbyopia. All concepts of the NAVQ-P were considered relevant to at least 30% of individuals with phakic presbyopia (Fig. 3). Twelve of the fifteen (n = 12/15, 80.0%) item concepts in the NAVQ-P were considered relevant to at least 50% of participants. The NVCI instrument was relevant to a total of 26 participants (n = 26/35, 74.3%). Assessment of relevance was not applicable to the NVCP, NVS, PGIS-Presbyopia and PGIC-Presbyopia instruments.

“Reading small printed text on paper such as newspaper—um, oh extremely difficult as I said before. Everything has to be large print.” (M50-MOD-R2-US8)

The concepts listed below demonstrated lower relevance (relative to other items) for participants with phakic presbyopia. Participants either did not report difficulty with the described visual activity in relation to their presbyopia or did not perform the task in the past seven days (Fig. 3). These items were retained, pending psychometric evaluation, but were flagged as potential candidates for deletion at a later stage.

- Reading on a tablet device: n = 15/35 (42.9%) participants reported as relevant.
- Seeing things in bright light: n = 11/35 (31.4%) participants reported as relevant.
- Ability for the eye to adjust between different vision fields: n = 8/18 (44.4%) participants reported as relevant.

“I don’t have difficulties to adjust. Because in any way, I’m blurry from a distance, I’m blurry at close distance so...” (F58-MOD-R2-FR2)

### General feedback on the instruments from round 1 and round 2 interviews with individuals with phakic presbyopia (n = 35)

No important concepts were identified as missing by participants. Participants were asked about the usability of the ePRO, with most participants (n = 11/15, 73.3%) who were asked in round 2 (stage 5) confirming that the text size was adequate, and n = 15/17 (88.2%) reporting no concerns with navigation on the device. A small number of participants (n = 3/35, 8.6%) estimated how long it would take them to complete the instruments, which ranged from 10 (n = 2/3, 66.7%) to 20 min (n = 1/3, 33.3%). However, these estimations may be inflated given that the instruments were completed during the interview which involved discussing the response to each item following completion.

“I think I would have needed 10 min.” (F44-MILD-R2-DE3)

## Discussion

To address the need for a PRO that assesses phakic presbyopia near vision functioning, the present study details the modification and content validity testing of the NAVQ-P and additional instruments (NVS, NVCI, NVCP, PGIS-Presbyopia and PGIC-Presbyopia). Findings suggest that the NAVQ-P demonstrates content validity as an assessment of near vision functioning which reflects the most important concerns of individuals with phakic presbyopia. The additional instruments are appropriate to assess near vision correction independence (NVCI), near vision correction preference (NVCP), and near vision satisfaction (NVS). The study involved the successful modification of the original NAVQ to address the limitations of the use of this instrument in clinical studies with individuals with phakic presbyopia.

The study had been designed in line with best practice and standards outlined in regulatory guidance on the steps necessary to establish content validity and feedback from the FDA was obtained [21, 24, 25, 30]. As a result, the NAVQ-P is a product of rigorous research involving an initial literature and instrument review and social media listening, multiple rounds of combined qualitative CE and CD interviews (with individuals with phakic presbyopia and HCPs) and engagement with regulators. Findings from an initial literature and social media review informed revision of the original NAVQ [16, 22]. The NAVQ-P was then subject to CD with individuals with phakic presbyopia and mapped alongside findings from qualitative CE interviews (described elsewhere) [23] to ensure all important concepts were included. The concepts included in the NAVQ-P also reflect findings from previous literature investigating the individual experience of presbyopia [8, 10–12, 14, 31]. The two rounds of CD interviews allowed for modifications to be implemented to the instrument following round 1 interviews, which could then be evaluated during round 2 interviews.

Participants demonstrated good understanding of the NAVQ-P item wording, recall period and response options in round 2 (stage 5) interviews. The three new items that were added following round 1 CD interviews and FDA feedback were well understood and had reasonably high relevance to presbyopic participants; supporting inclusion in the NAVQ-P. A small number of modifications were made following round 2 based on the CD findings to enhance interpretation. Additionally, formatting changes were made to enhance comprehension. HCPs confirmed that the NAVQ-P concepts were clinically relevant to individuals with phakic presbyopia.

As the NAVQ-P and additional instruments were developed and cognitively debriefed in an electronic mode of administration (on a tablet device), the interviews confirmed usability of the ePRO and that the font size of an electronic administration of instruments was appropriate for individuals with phakic presbyopia. In addition, throughout the development of the NAVQ-P it became apparent that individuals who experienced both myopia and presbyopia used their glasses differently to see up close. Those with comorbid myopia took their long-distance glasses off to see up close, while those with phakic presbyopia only put their reading glasses on to see up close. With this in mind, the instructions were updated to specifically ask participants to think about when they are not wearing their glasses to see things close to them. As a result, the instrument is suitable for use in individuals with phakic presbyopia who do or do not have comorbid myopia.

A key strength of this study was that clinical relevance and development of the instruments was ensured via collaboration with the NAVQ developer and specialist HCPs. Regulatory advice guided further development of the NAVQ-P and NVCI instruments and was pivotal in ensuring that the NAVQ-P and NVCI meet the quality standards for content validity, supporting use of these instruments as clinical trial endpoints with potential to support label claims. The NAVQ-P provides a unique opportunity to assess near vision functioning that is specific to individuals with phakic presbyopia. Other PROs commonly used in ophthalmology populations to assess visual function (such as the NEI RQL-42 or the NEI VFQ-25) assess some concepts that are not relevant to presbyopia, such as ability to do distance vision activities or limitations with peripheral vision, have been developed with individuals who have received refractive eye surgery, or require individuals to respond to items thinking about their vision when using vision correction [19, 20].

The study sample included individuals with phakic presbyopia from multiple countries (US, Germany, and France) and quotas were used to ensure the sample had diverse demographic and clinical characteristics. This enables confidence that findings are representative of the wider phakic presbyopia population and provides evidence of cross-cultural validity, specifically in the US and Europe. Additionally, the HCPs from the US, France, Japan and Germany all confirmed clinical relevance of the NAVQ-P and additional instruments in these countries, further supporting cross-cultural validity and clinical relevance. A wide range of refractive error was included in the sample, with this study confirming the suitability of the use of the NAVQ-P and additional instruments in individuals with phakic presbyopia, regardless of their refractive error (i.e. severity).

While multiple countries were included, all were western countries in highly developed nations, therefore, further research in countries in Asia, South America and/or Africa in the future would be of interest to provide further insight regarding the degree to which the findings can be generalized cross-culturally.

## Conclusions

The study findings reported here support the content validity of the newly adapted NAVQ-P, NVCI, NVCP, NVS, PGIS-Presbyopia and PGIC-Presbyopia in individuals with phakic presbyopia. Psychometric evaluation is planned to support finalization of scoring (with possible item reduction), and assess the validity, reliability, and importantly ability to detect change over time for the instruments, ultimately confirming the adequacy of the NAVQ-P and additional instruments as clinical trial endpoints in support of potential labelling claims.

## Supplementary Information

**Additional file 1**. Additional information on methodology.

**Additional file 2**. Example HCP interview guide questions.

**Additional file 3**. Example interview guide questions with participants with presbyopia.

## Data Availability

The data that support the findings of this study are available from Novartis Pharma AG but restrictions apply to the availability of these data, which were used under license for the current study, and so are not publicly available. Data are however available from the authors upon reasonable request and with permission of Novartis Pharma AG.
