# Supplementary material for: Evaluation of the content validity of patient-reported outcome (PRO) instruments developed for use with individuals with phakic presbyopia, including the Near Activity Visual Questionnaire-presbyopia (NAVQ-P) and the near vision correction independence (NVCI) instrument
Source: J Patient Rep Outcomes. 2021 Oct 23;5:109. doi: 10.1186/s41687-021-00379-x (PMC8542063; doi:10.1186/s41687-021-00379-x)
Supplement: Supplementary file 1 — Additional file 1. Additional information on methodology. [file 41687_2021_379_MOESM1_ESM.docx]

## Appendix A. Additional information on methodology

### Case report form (CRF)

The CRF collected information from the participant’s ophthalmologist/optometrist pertaining to the participant’s clinical diagnosis of phakic presbyopia and included measurement of the participant’s visual acuity score when the participant was not using their near vision correction method (using the EDTRS [Early Treatment Diabetic Retinopathy Study] method or Snellen equivalent). The severity of presbyopia was determined using the participant’s binocular distance-corrected near visual acuity (DCNVA) at 40cm, with mild defined as a DCNVA worse than 20/40 but better than 20/80, and moderate-severe a DCNVA 20/80 or worse. Participant severity of Near Addition (Near ADD) was also assessed where mild was defined as +1.00D to +2.00D Near ADD, and moderate-severe defined as >+2.00D Near ADD.

### Demographic form

The participant demographic form was used to collect demographic information and details about participants’ experience of phakic presbyopia, including their self-reported impression of the severity of their presbyopia (on a four point scale from very severe to mild), and participant reported experience of myopia which was explained as ‘nearsightedness’ in the screener form (yes or no options). Only myopia was asked (rather than other co-morbid conditions e.g. hyperopia) as it was acknowledged that co-morbid myopia can influence the way that presbyopia is corrected, compared to participants who do not have co-morbid myopia.

### Eligibility criteria

Participants were required to be aged 40-65 years with clinician-confirmed diagnosis of phakic presbyopia. The minimum age of 40 years was implemented as this is the age that presbyopia commonly occurs.[1] The upper age limit reflects when an individual would no longer have any residual dynamic accommodation and when media opacities become more common, confounding the visual loss at near from presbyopia. All were required to be fluent, literate, and able to read and write, in the language of the country of recruitment (participants were not excluded from the study if they had difficulty reading due to their visual impairment). All participants were required to provide written informed consent and willing and able to participate in an in-depth interview. Participants were excluded if they had: a history of lens extraction/replacement; prior diagnosis of any other ocular condition (excluding myopia or hyperopia) that has an impact to visual acuity; or a physical/mental illness clinically considered to impact study engagement, input, and performance. Further information regarding the study inclusion and exclusion criteria can be found in Table 1.

Table 1. Eligibility criteria

| Eligibility criteria |  |
| --- | --- |
| **Inclusion criteria** | Participants diagnosed with phakic presbyopia aged 40-65 years with confirmation of diagnosis by an optometrist or other healthcare professional |
|  | Fluent speaker, literate and able to read and write in their local language |
|  | Willing and able to provide written informed consent and to perform all study activities; including participating in at least one in-depth interview. |
| **Exclusion criteria** | Participant had a history of lens extraction or replacement (e.g. cataract surgery). |
|  | Participant had a diagnosis of any other ocular condition, other than short-sightedness (myopia) or long-sightedness (hyperopia), that has an impact on visual acuity (e.g. cataracts). |
|  | Participant has any other physical or mental illness that, in the opinion of the recruiting clinician, might influence the responses they give during the interview or might impact the participant’s ability to engage with the interview or provide appropriate input. |

Key recruitment quotas were implemented to ensure representation of important clinical (severity of condition, co-morbid myopia [near-sightedness]), correction method and demographic (age, gender, educational attainment, race) subgroups. This ensured a diverse population (e.g. in terms of severity, education level) and thus that content validity would be evaluated across a range of individuals with phakic presbyopia.

### Ethical conduct

Institutional Review Boards (IRBs) provided ethical oversight for the US (Copernicus Group Independent Review Board (CGIRB); IRB ref: ADE1-18-049) and Germany (Salus IRB; IRB ref: Novartis CUNR844A2001), prior to study related activities. In France a letter was submitted to the Conseil National de l'Ordre des Médecins (CNOM), outlining study procedures and planned payments to recruiting health professionals. All participants provided written and verbal informed consent prior to taking part in an interview and were informed of their right to withdraw permission to use and disclose health information at any time. Participants were given a unique identification code and participant names and other identifiable information was removed from all transcripts and other documents. Participant data was pseudonymized for internal analysis and anonymized for the purposes of external publication.
