# Supplementary material for: Evaluation of the content validity of patient-reported outcome (PRO) instruments developed for use with individuals with phakic presbyopia, including the Near Activity Visual Questionnaire-presbyopia (NAVQ-P) and the near vision correction independence (NVCI) instrument
Source: J Patient Rep Outcomes. 2021 Oct 23;5:109. doi: 10.1186/s41687-021-00379-x (PMC8542063; doi:10.1186/s41687-021-00379-x)

## Appendix B. Example HCP interview guide questions


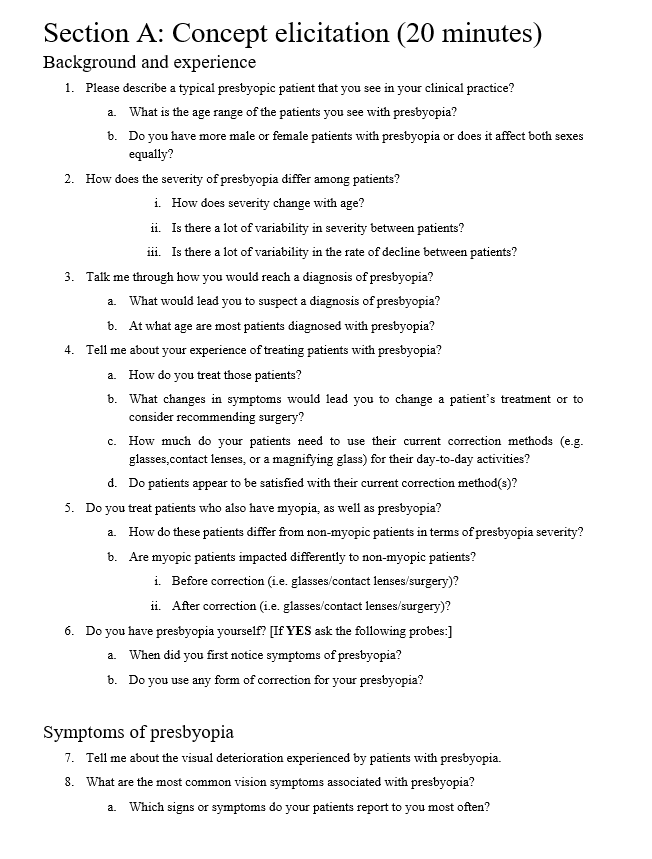


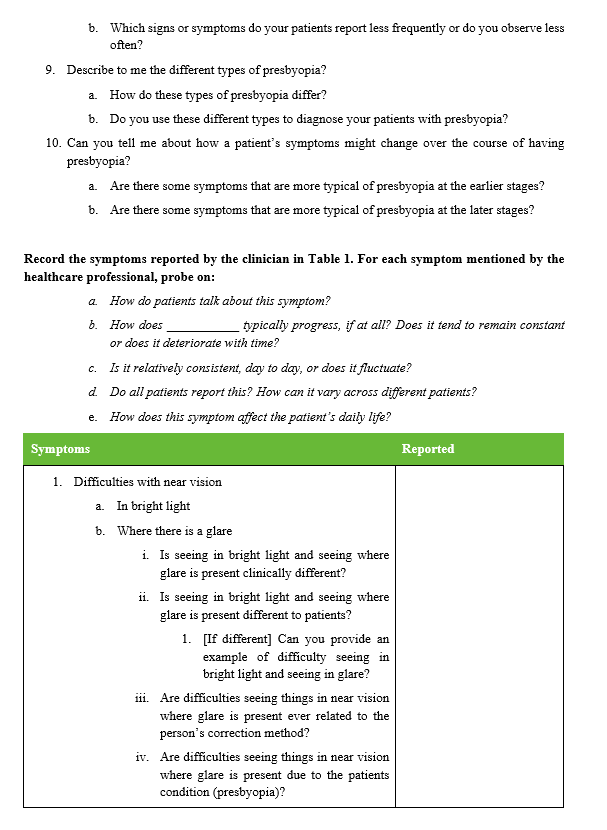


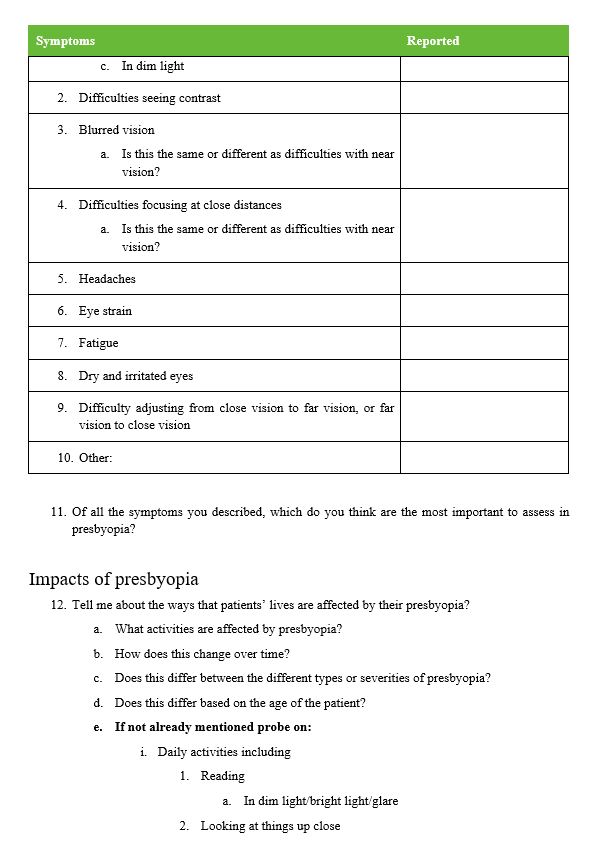


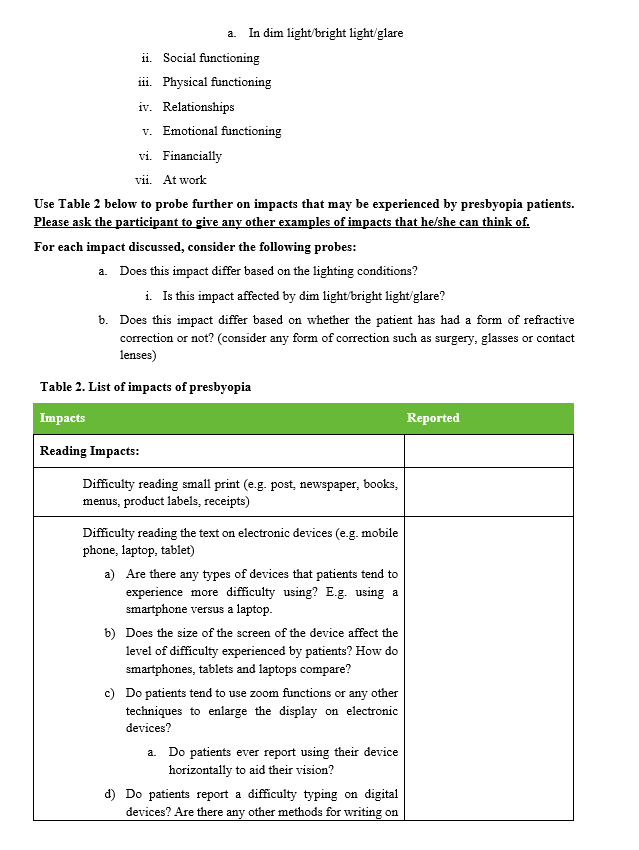


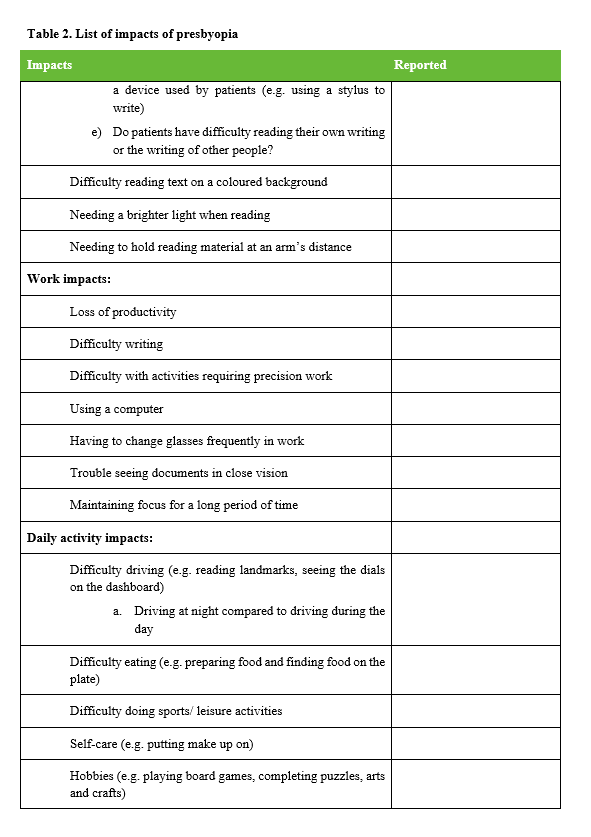


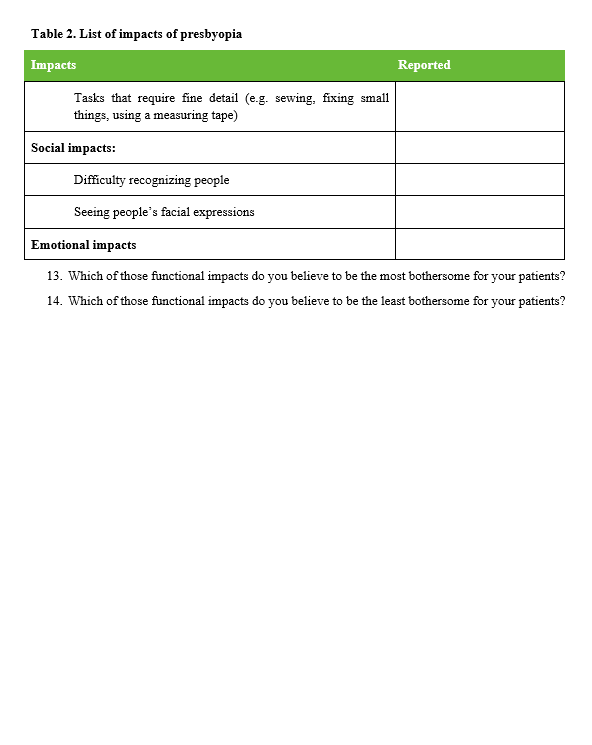


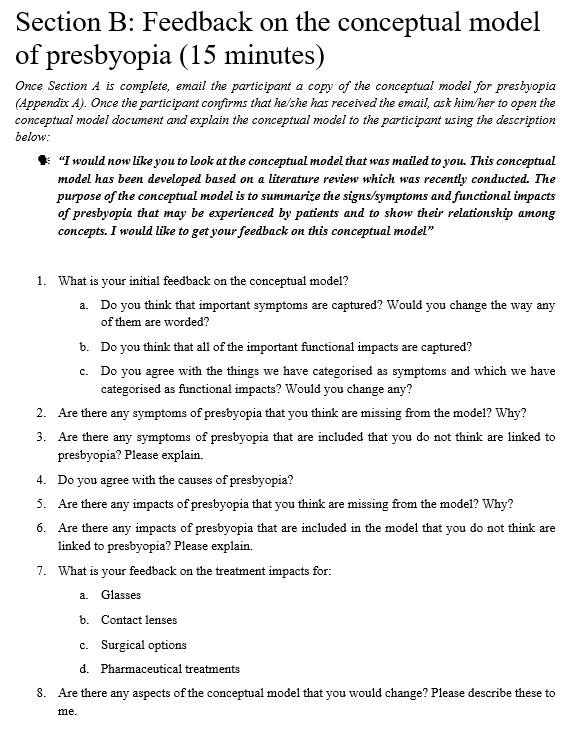


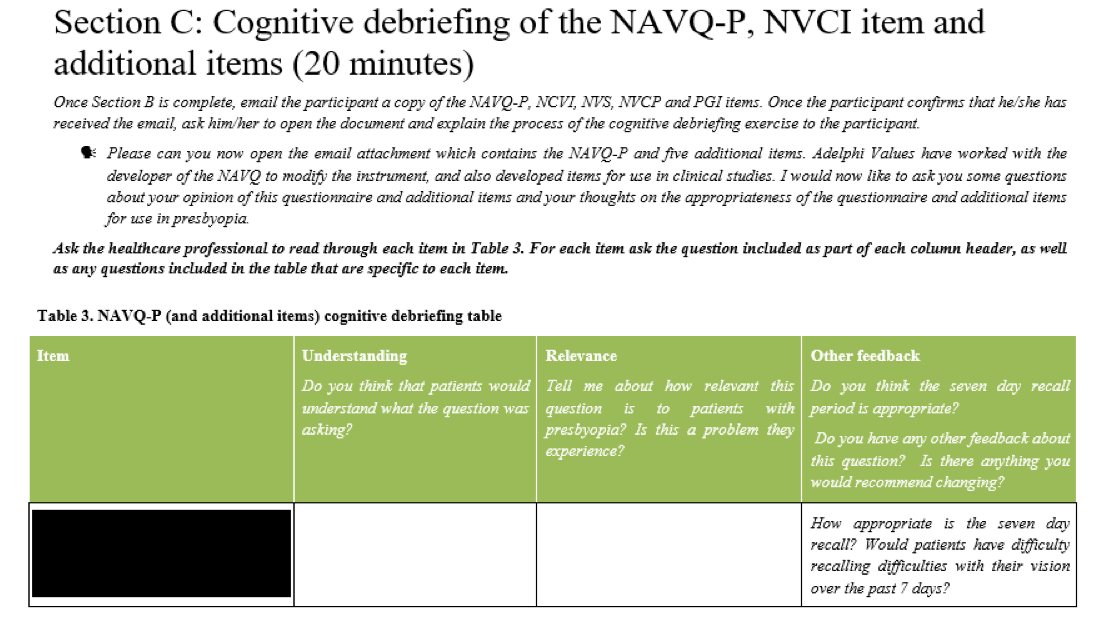


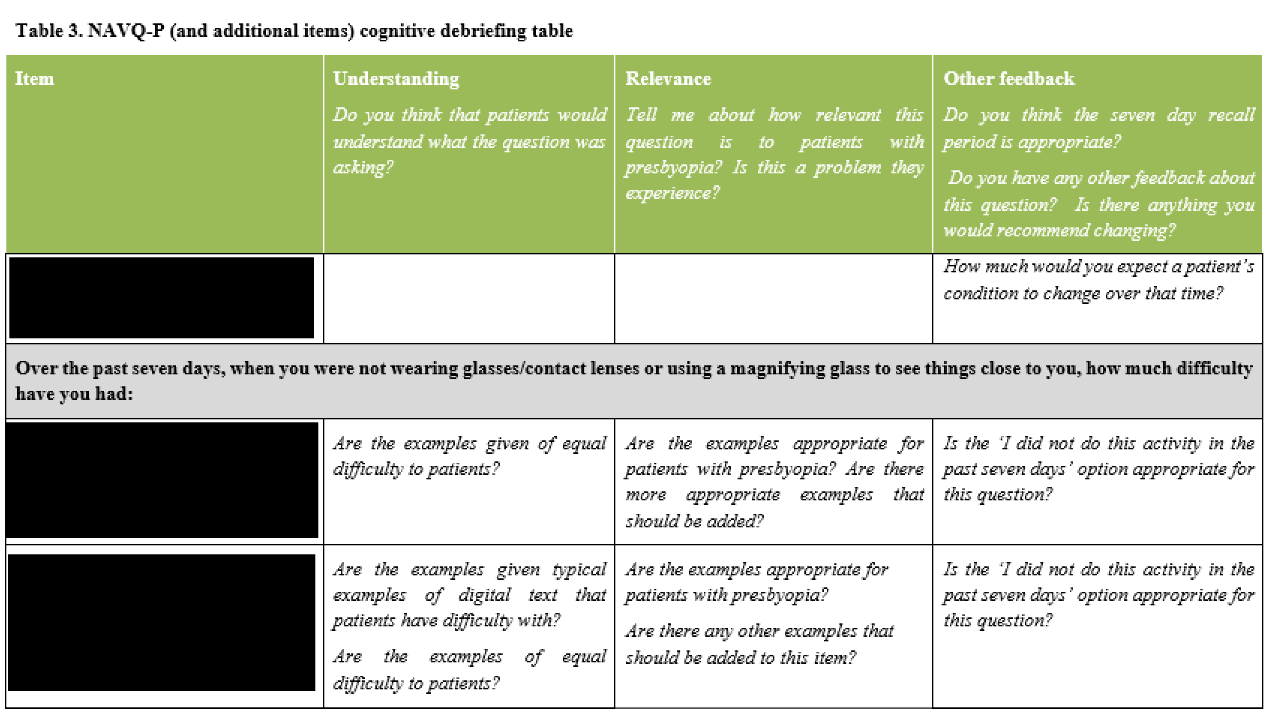


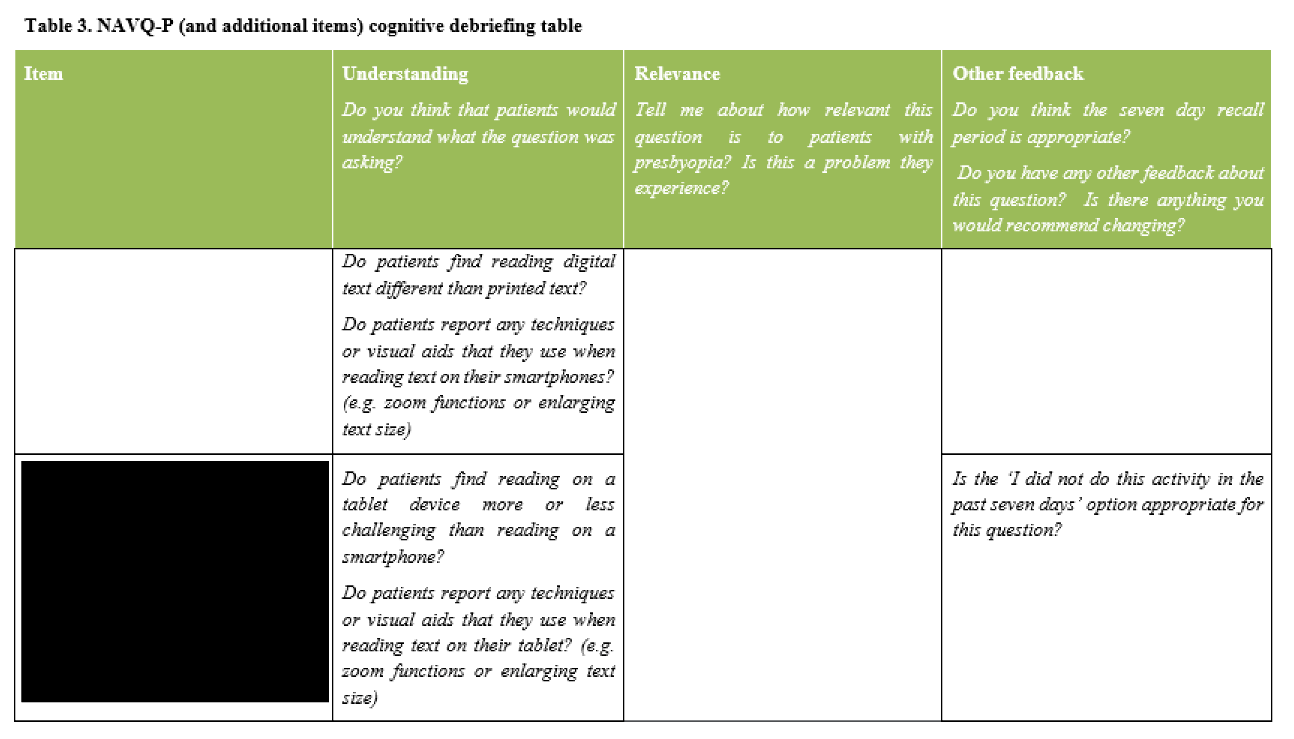


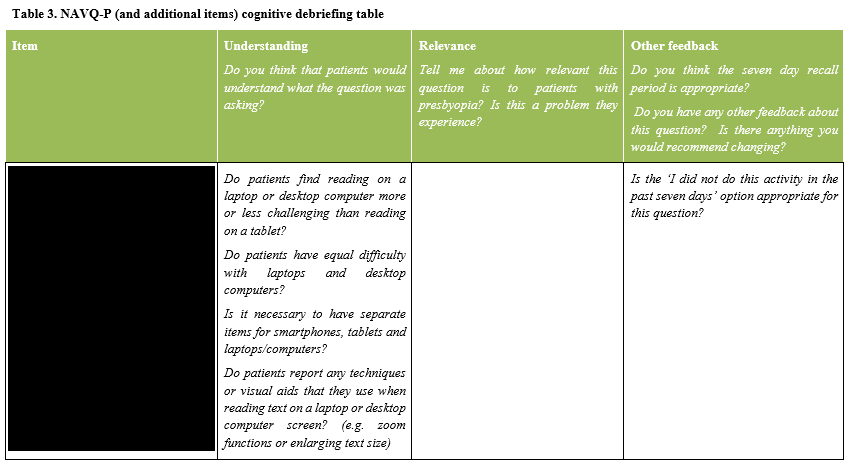


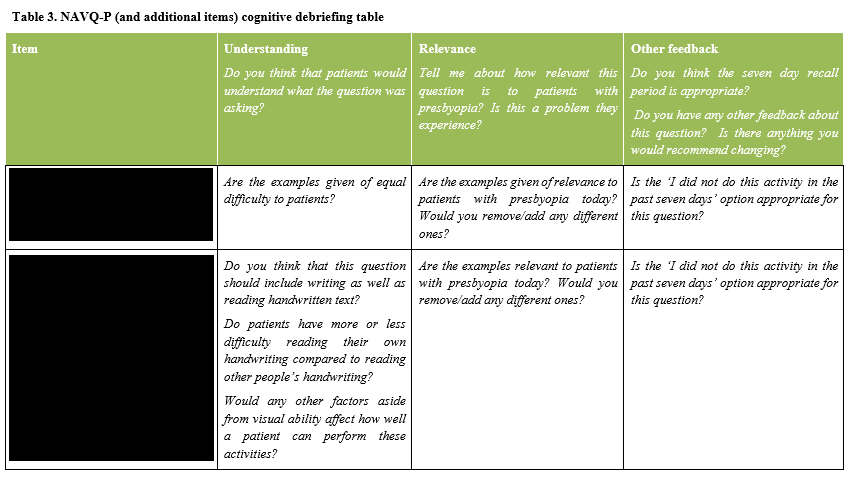


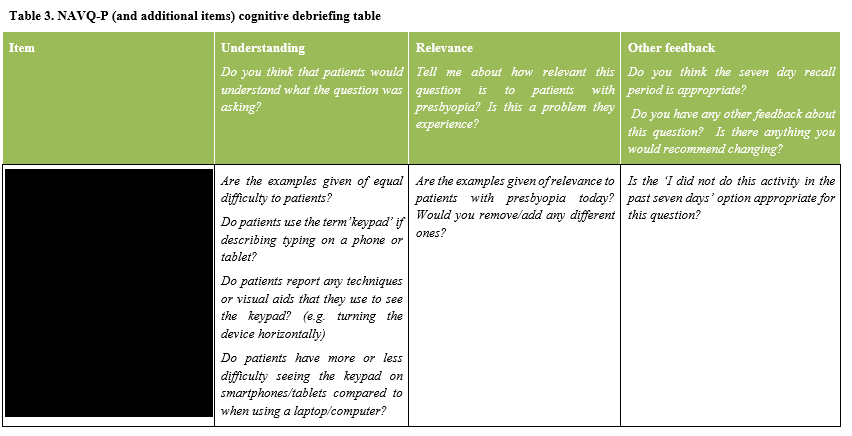


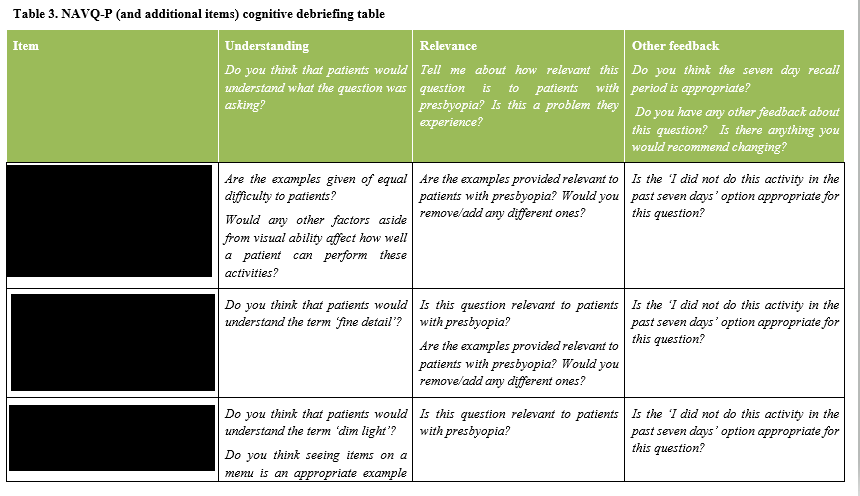


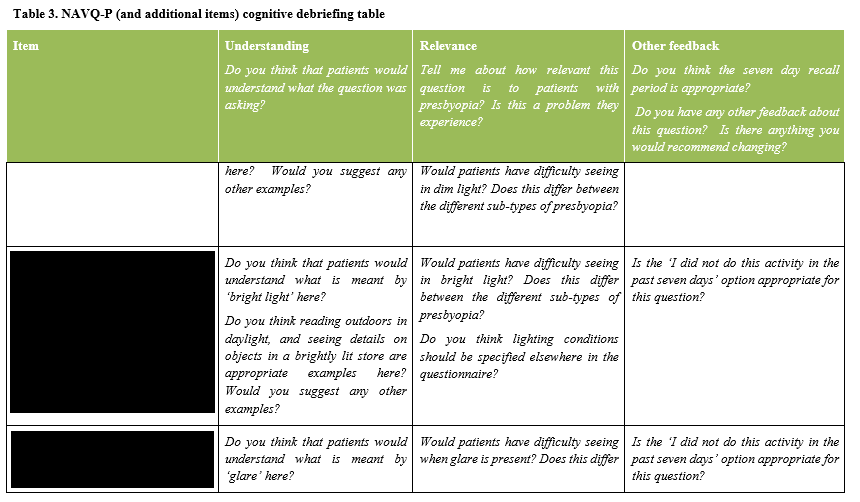


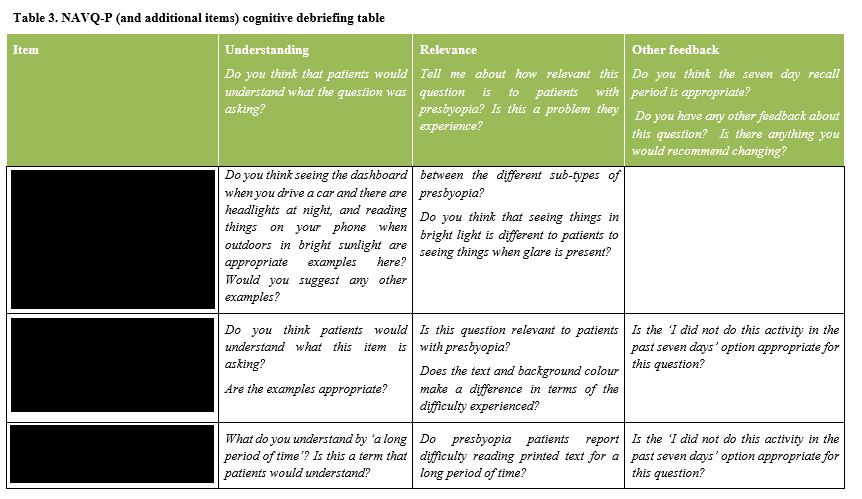


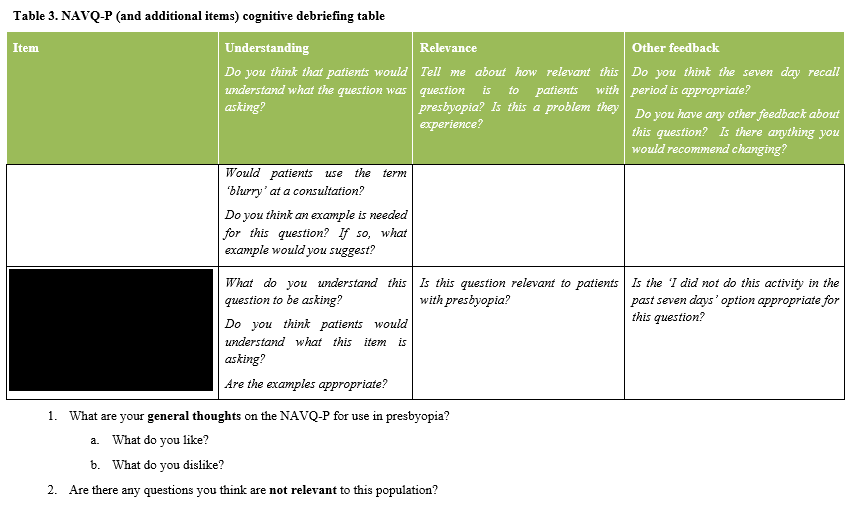


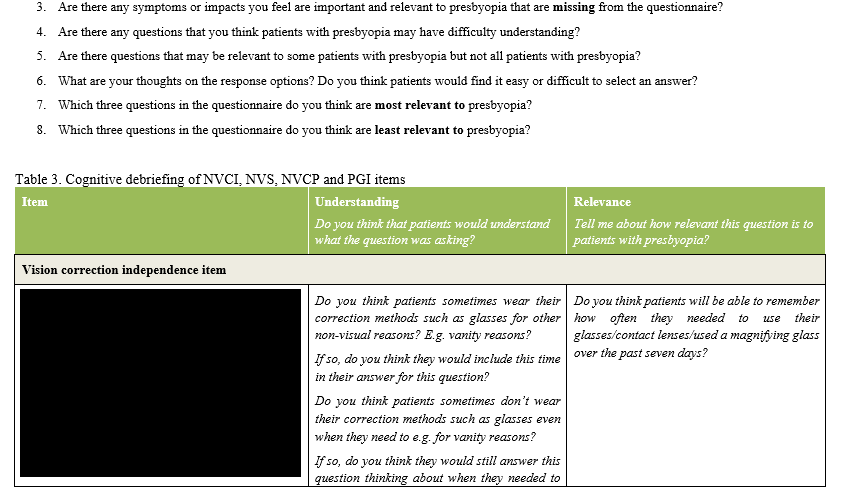


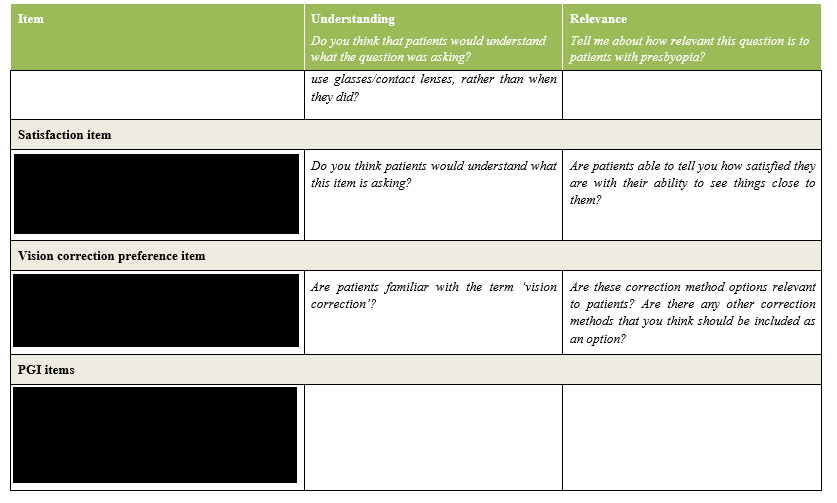


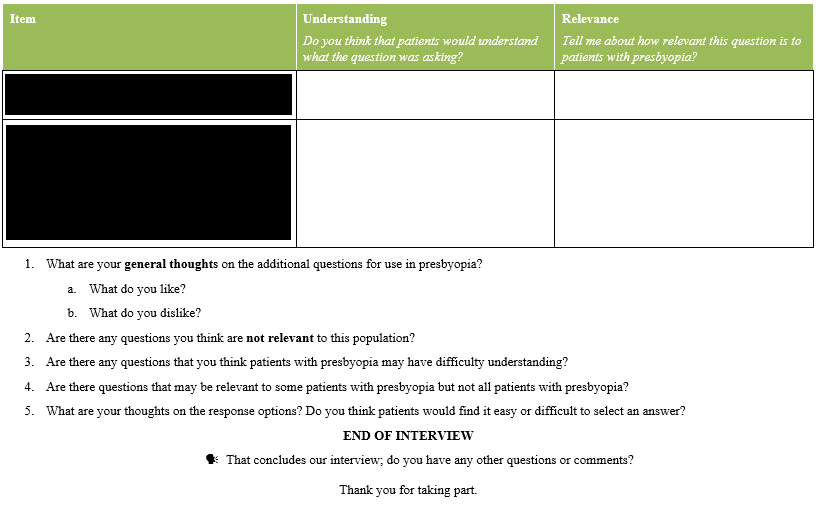


## Appendix C. Example interview guide questions with participants with presbyopia


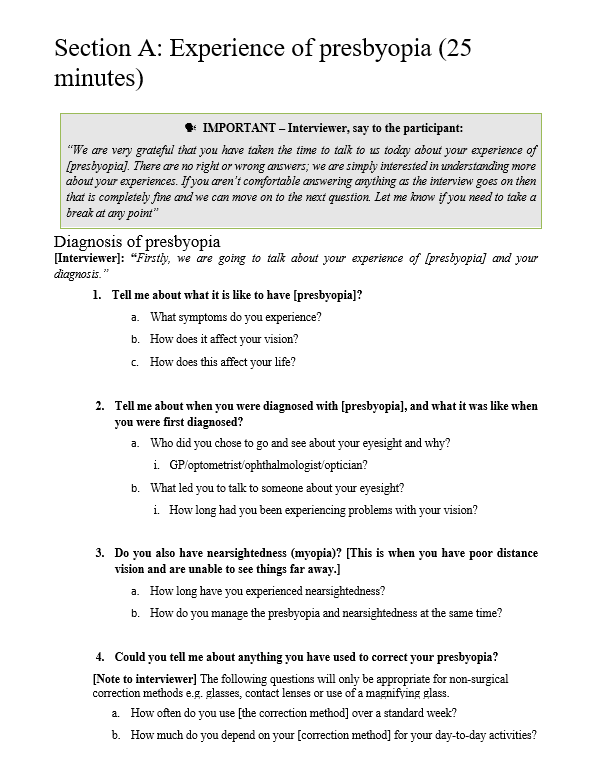


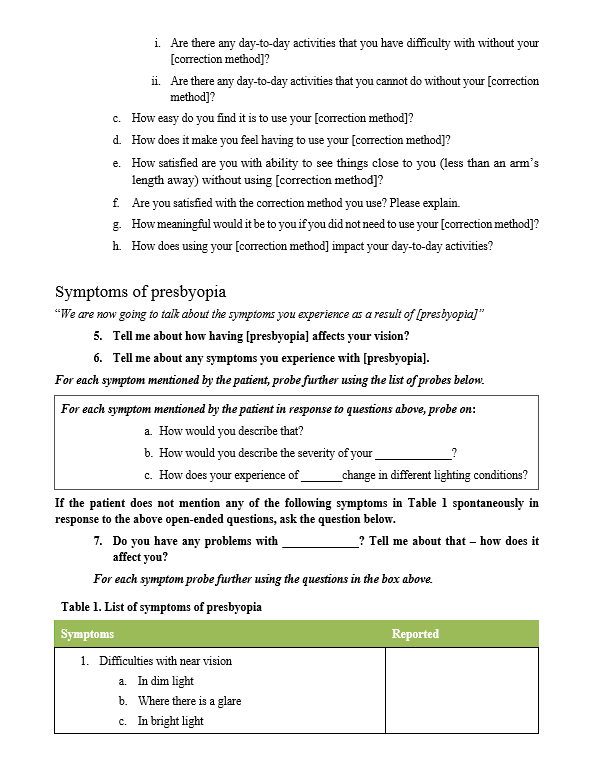


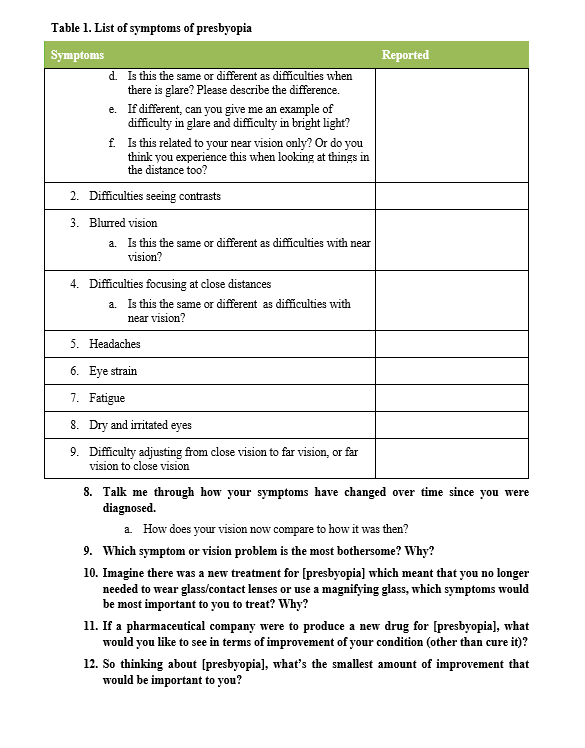


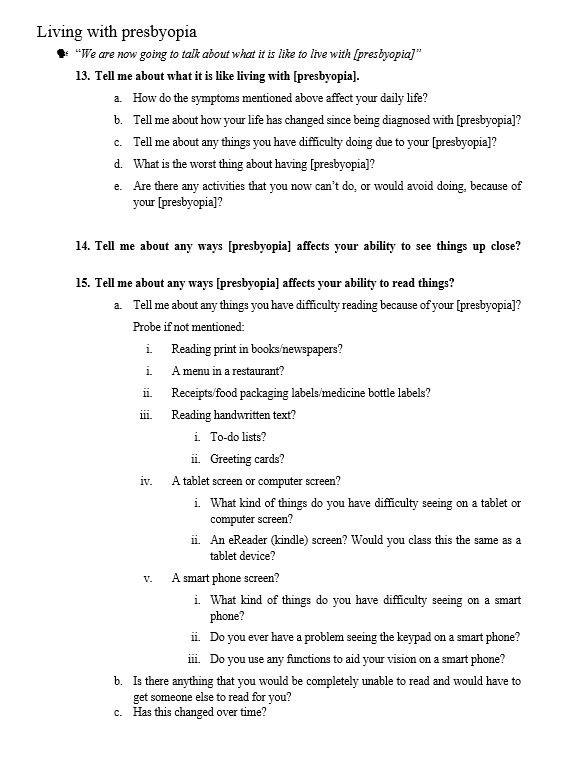


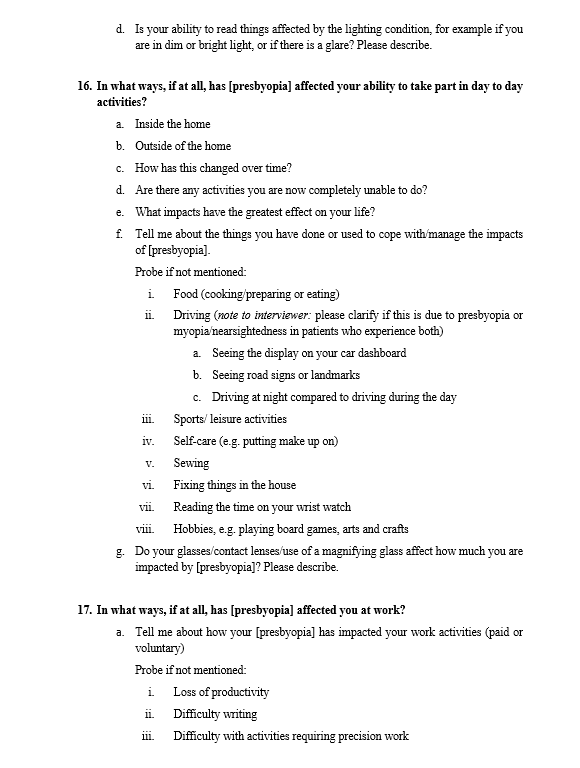


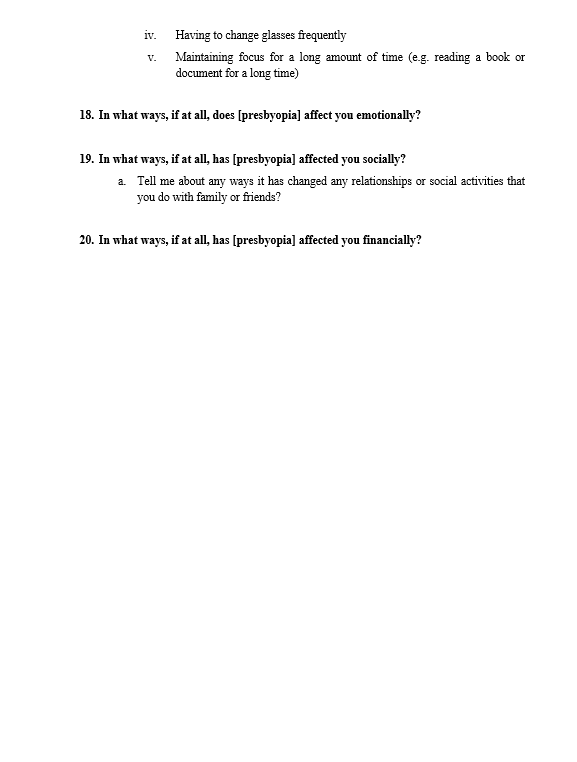


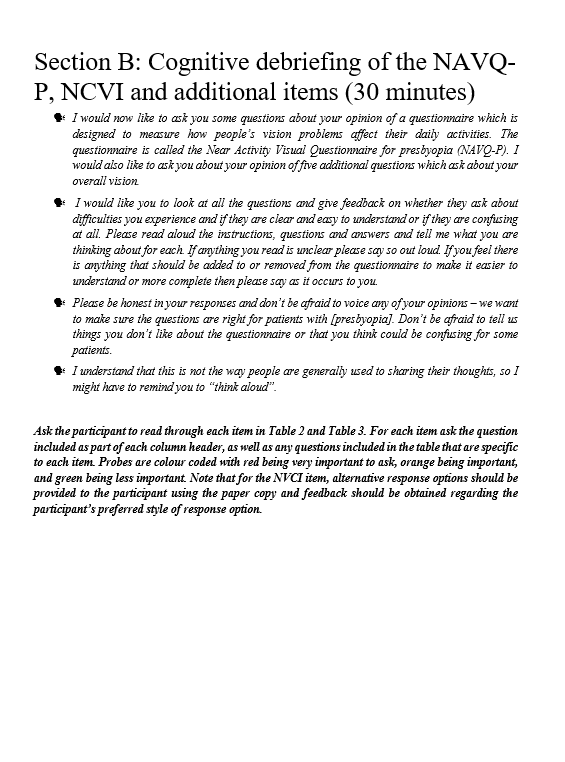


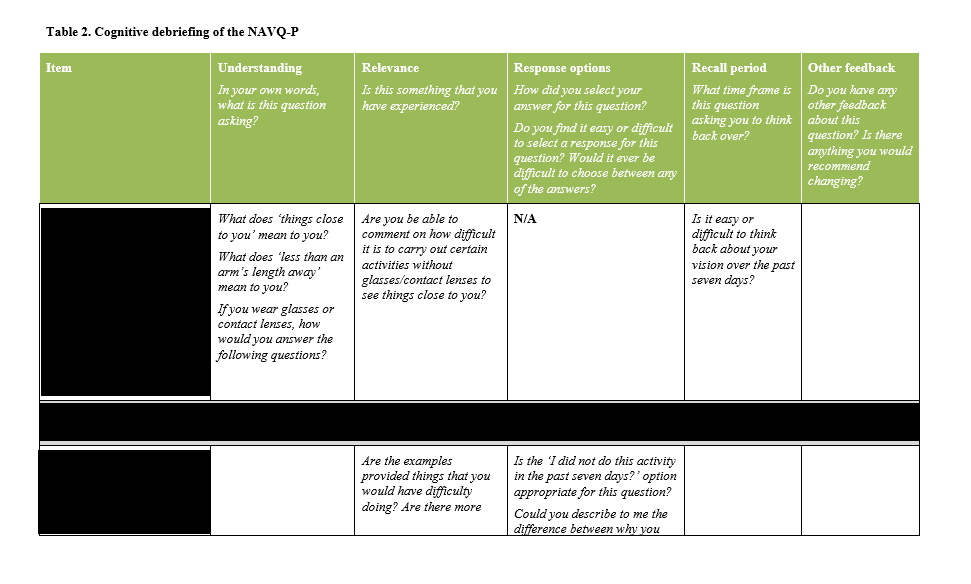


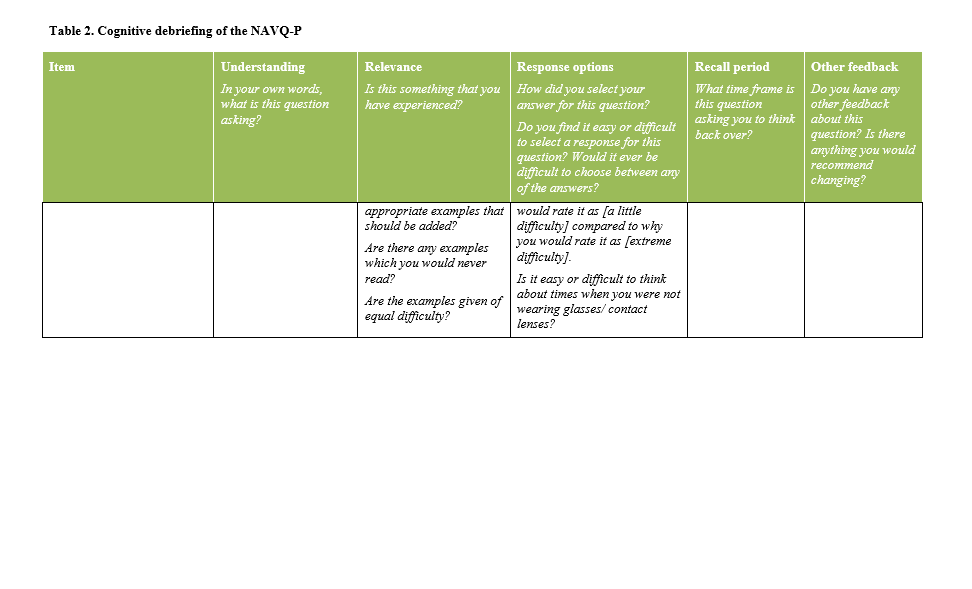


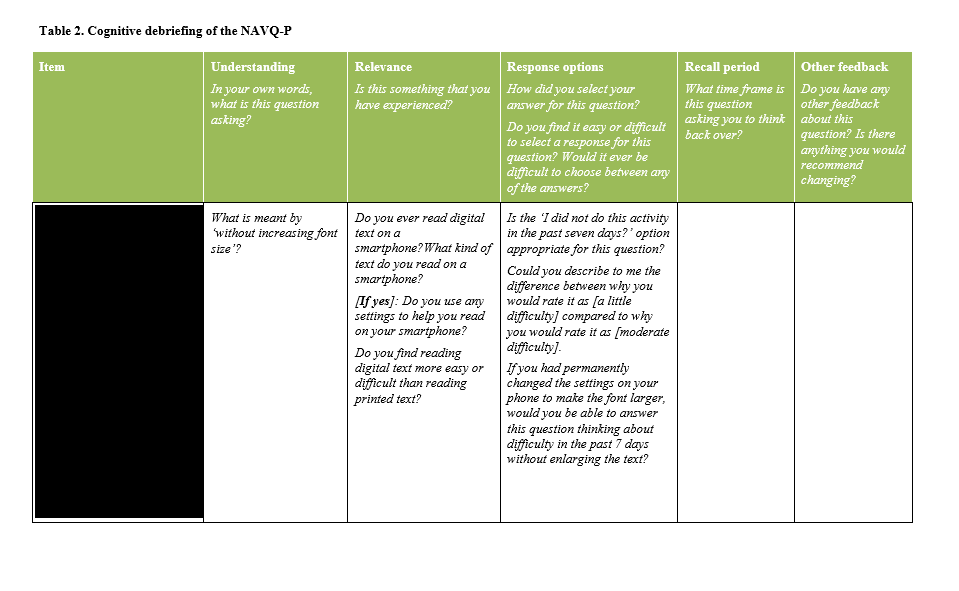


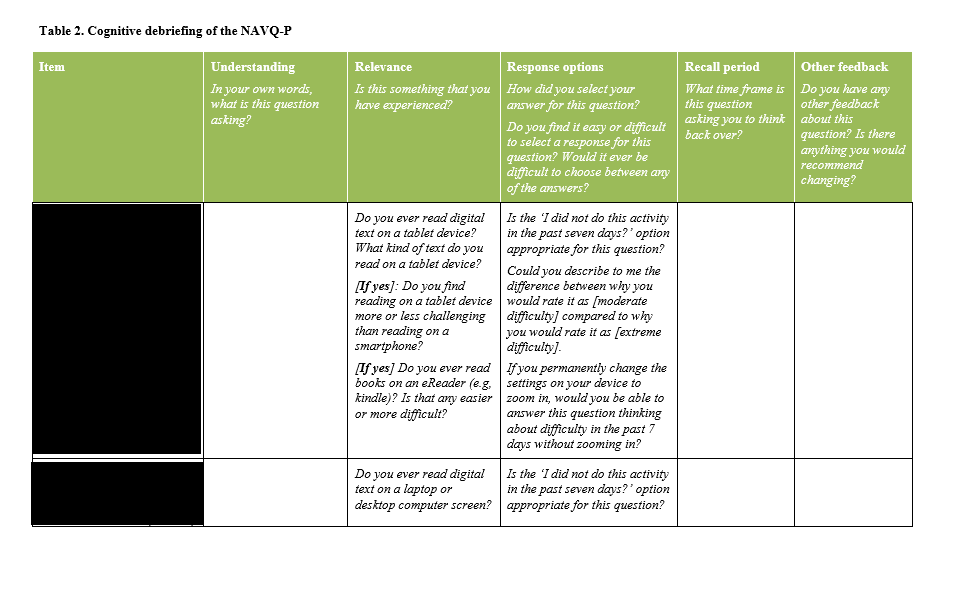


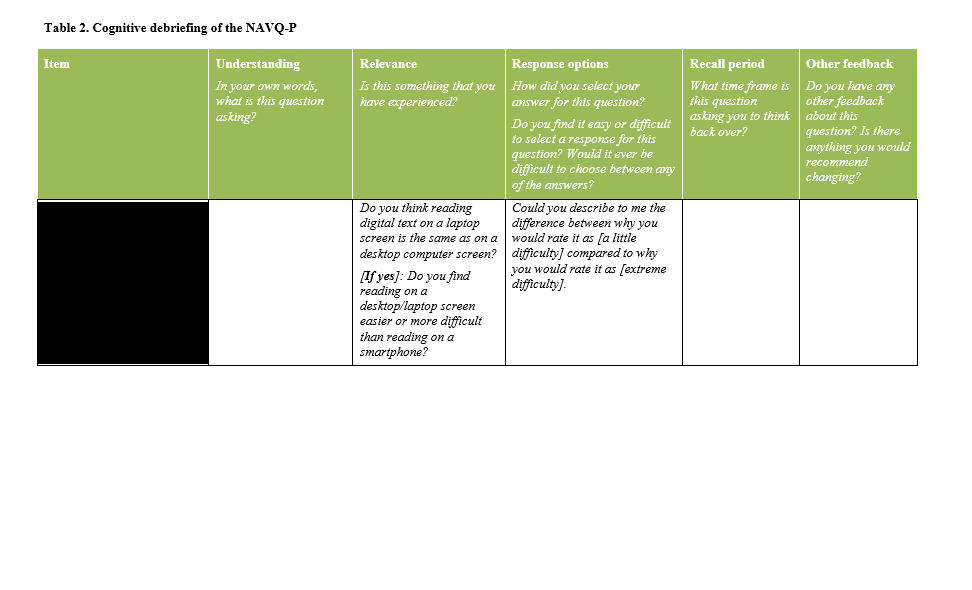


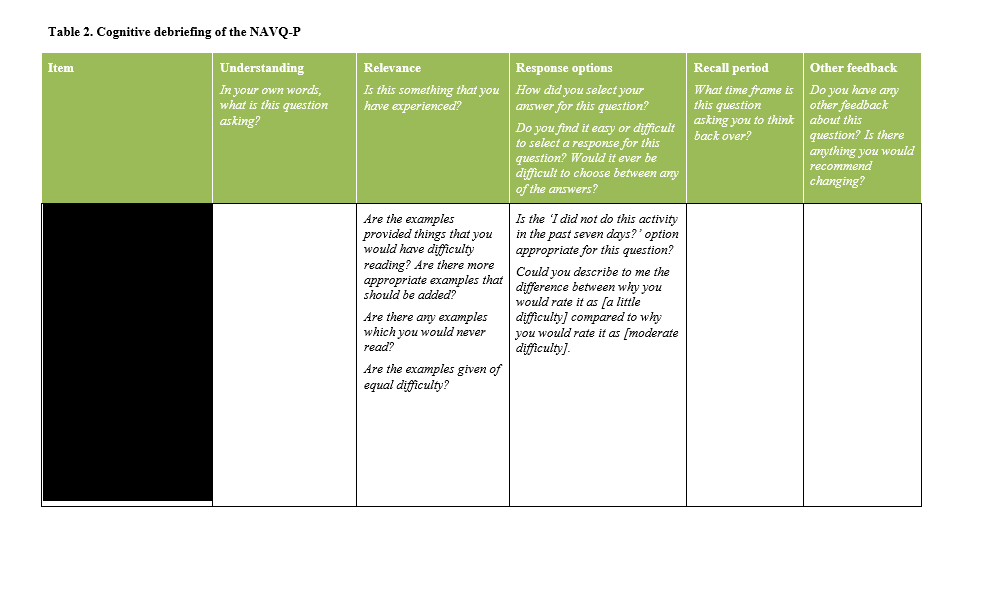


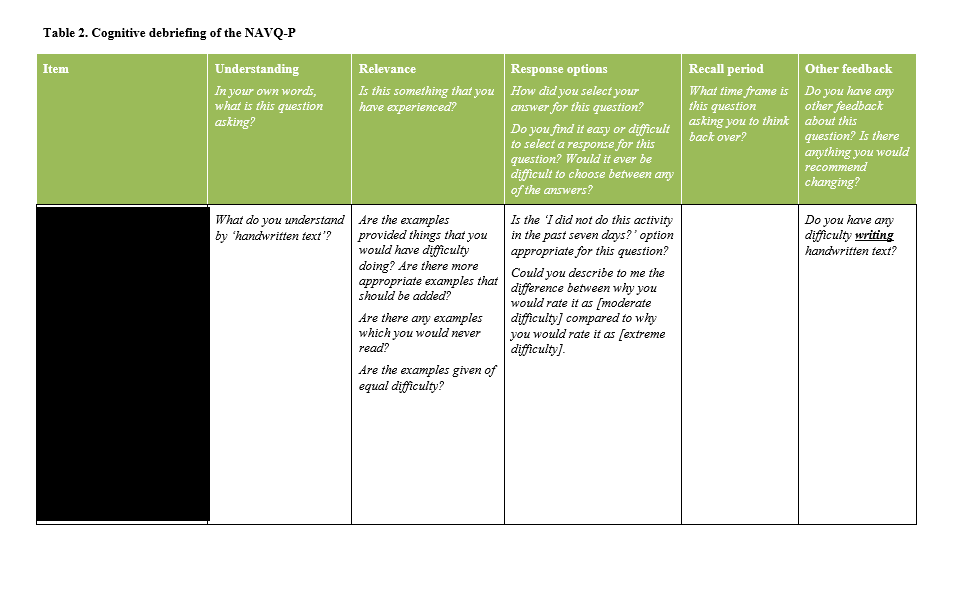


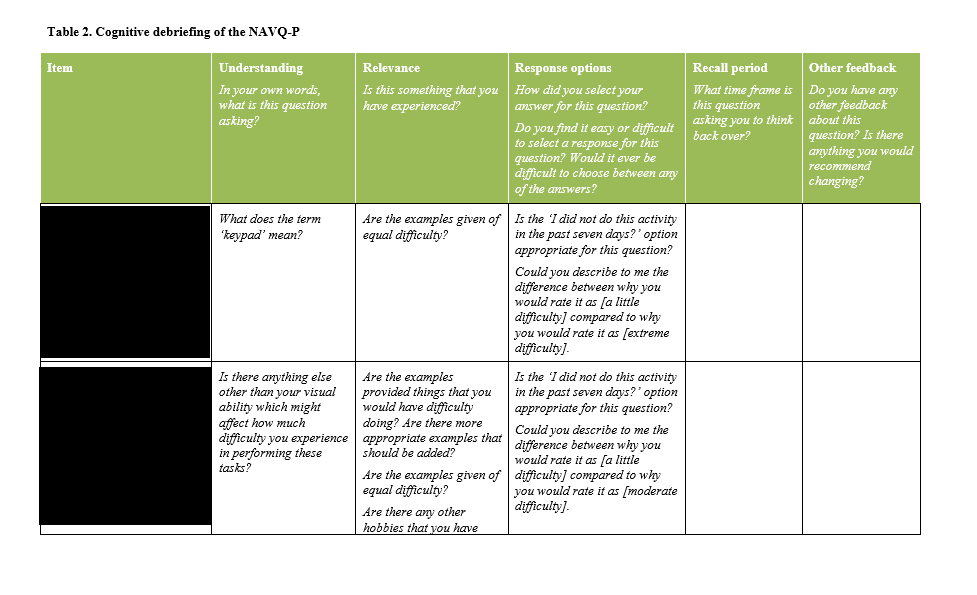


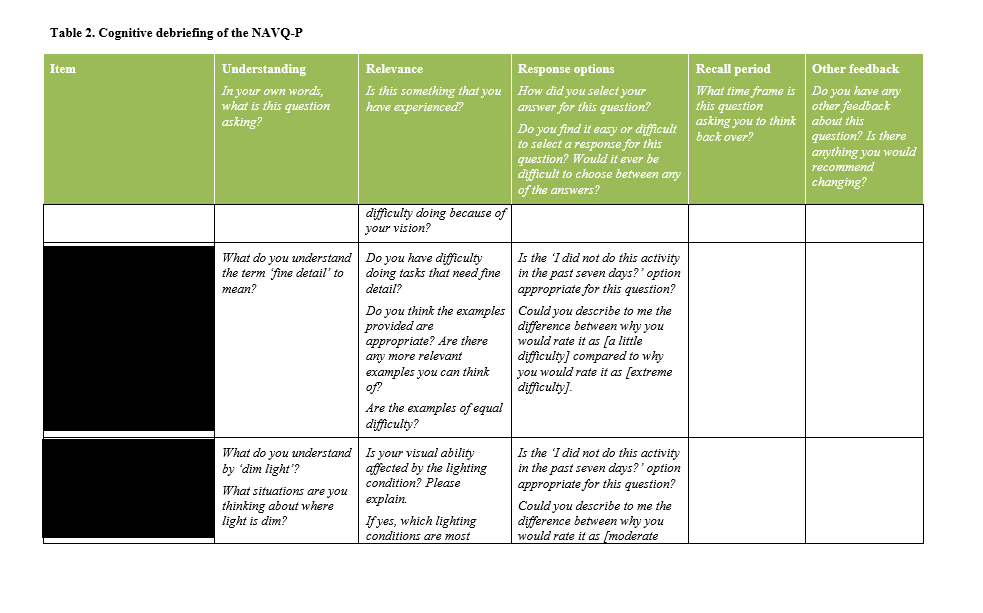


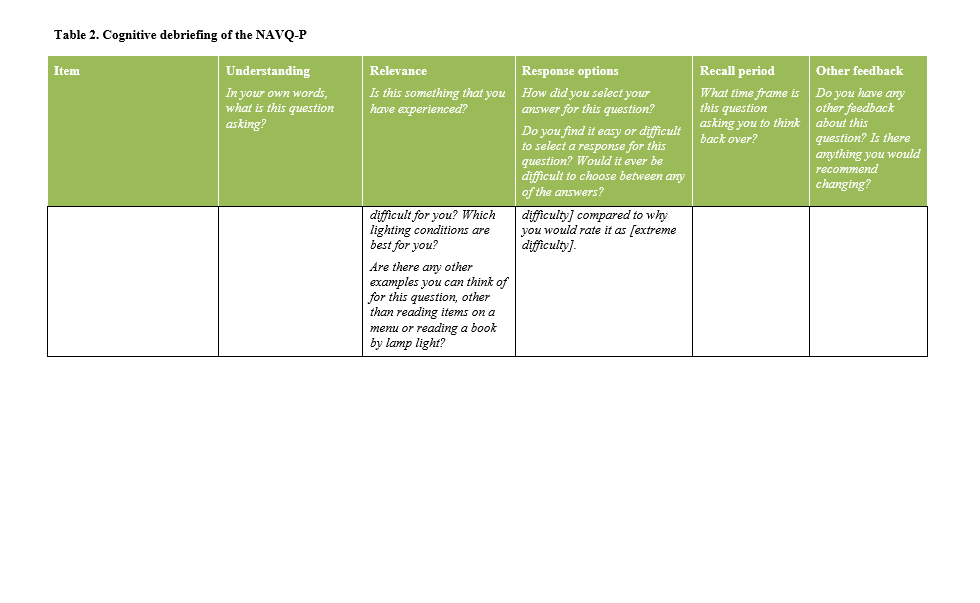


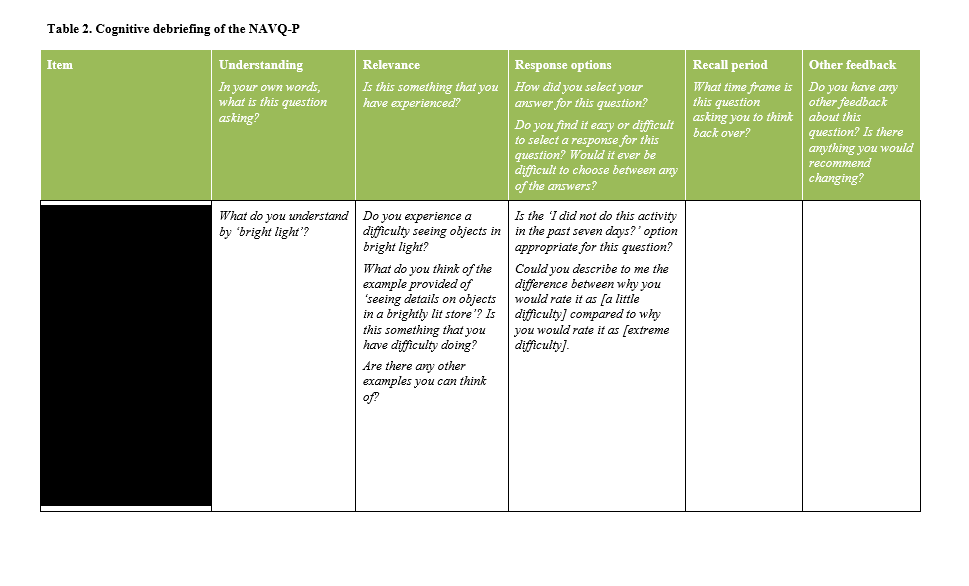


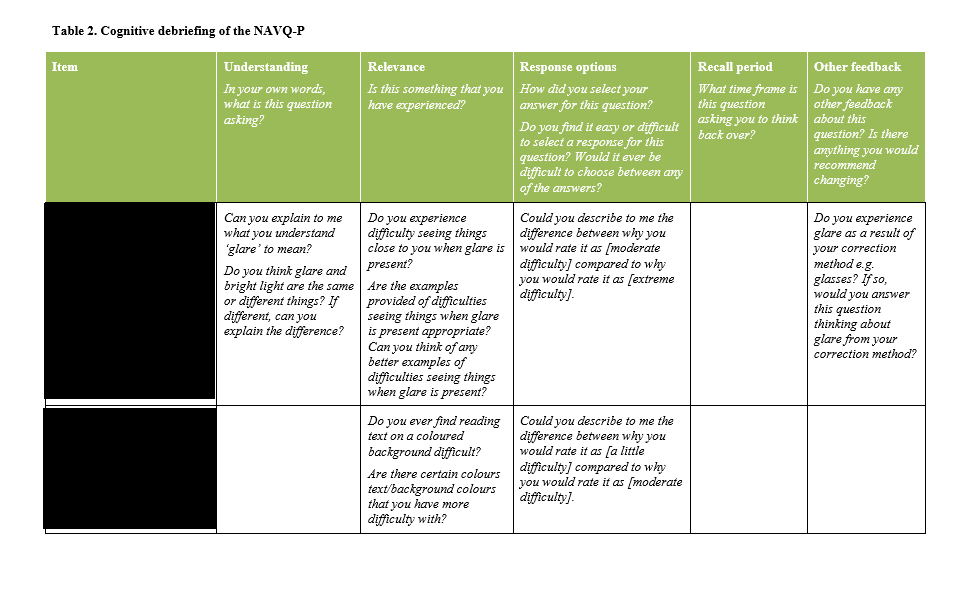


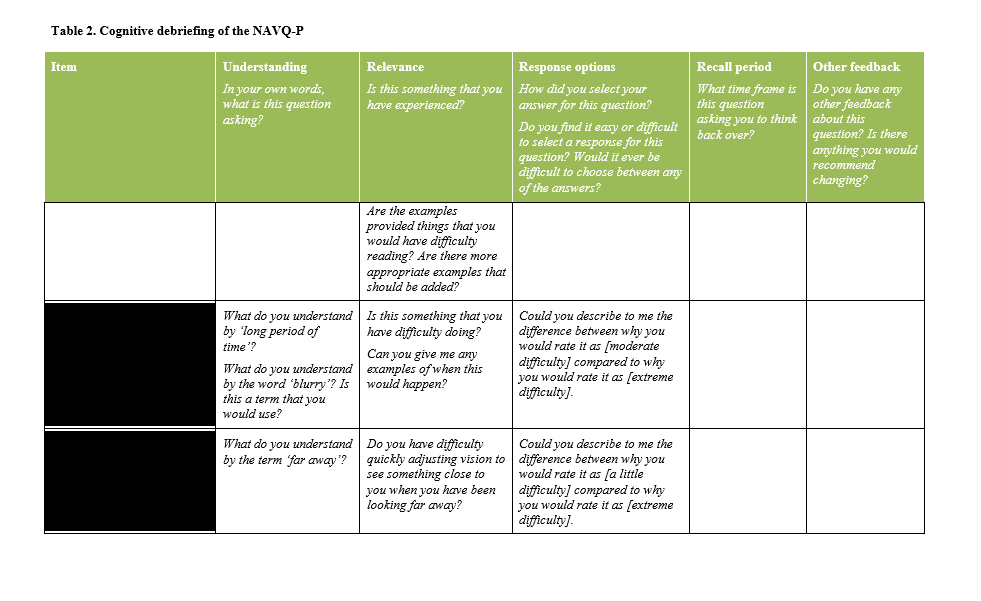


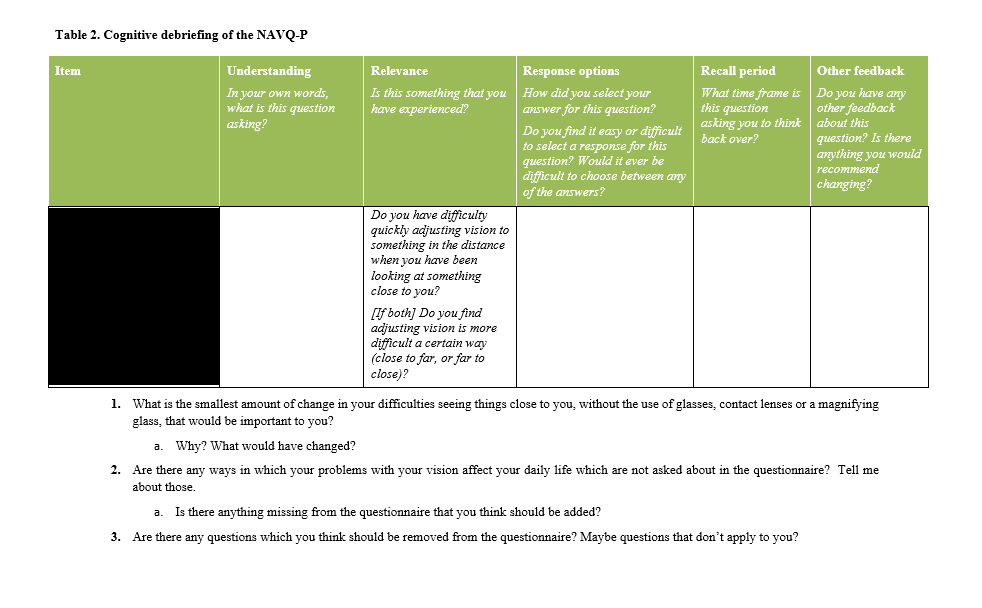


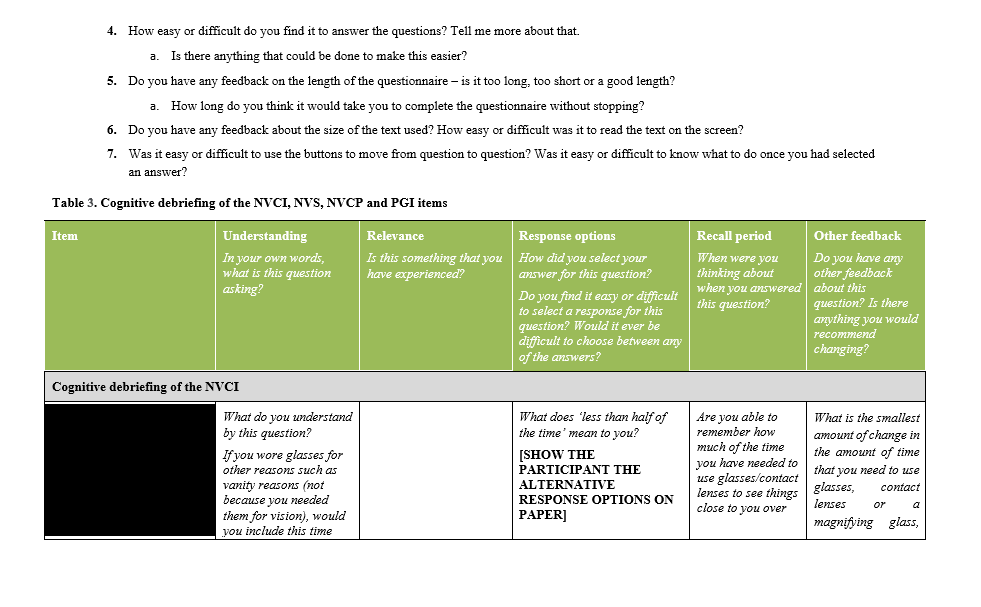


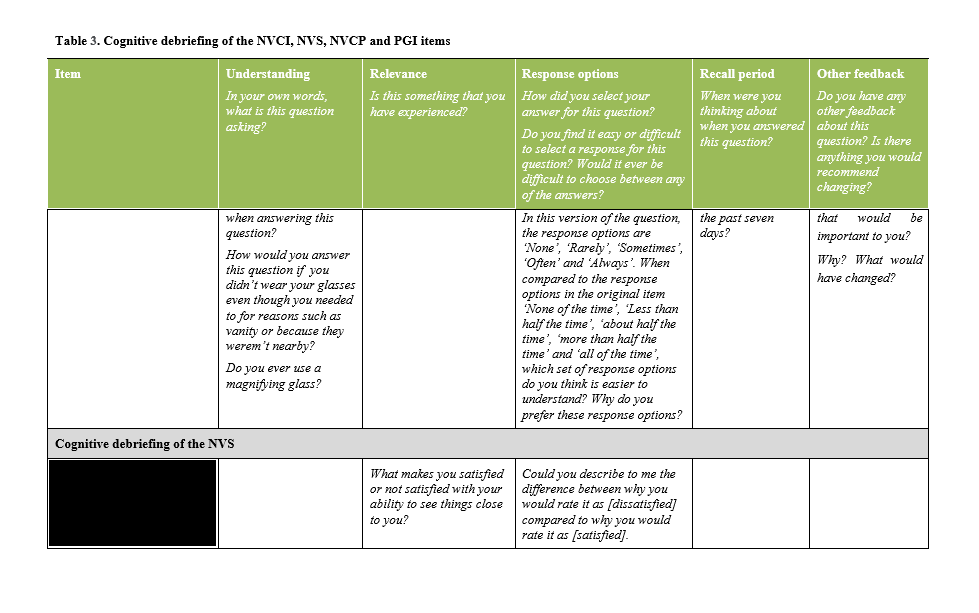


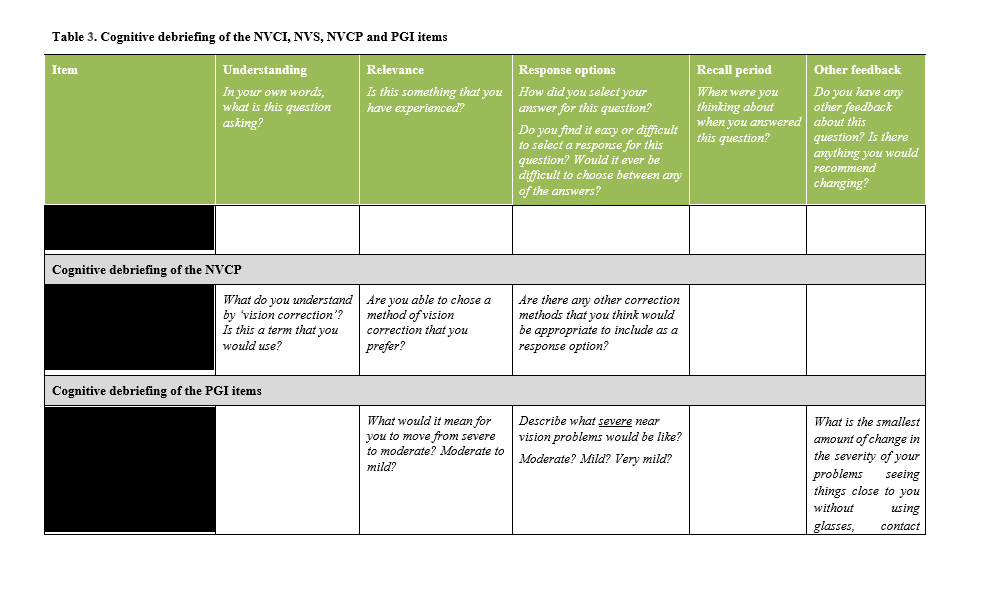


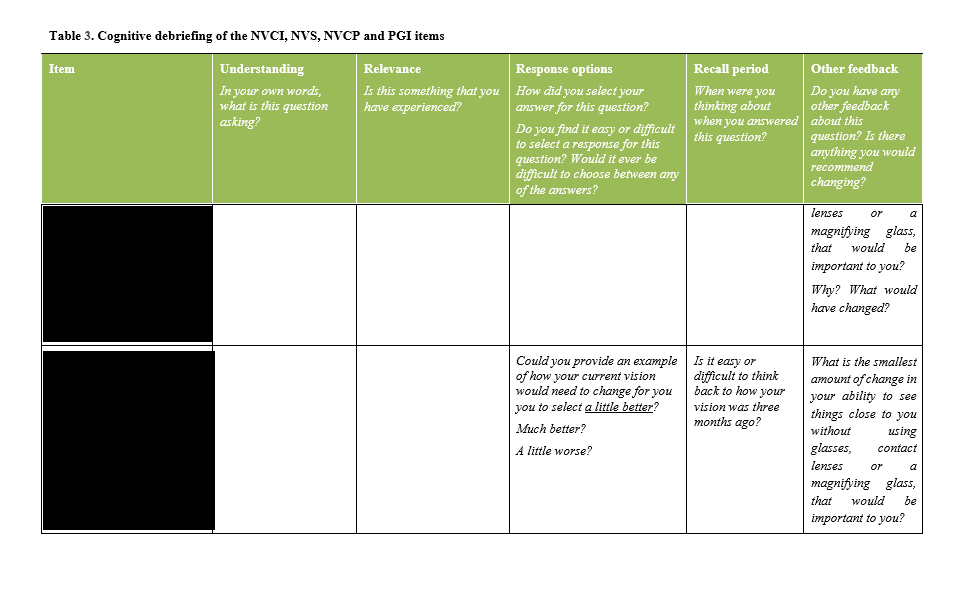


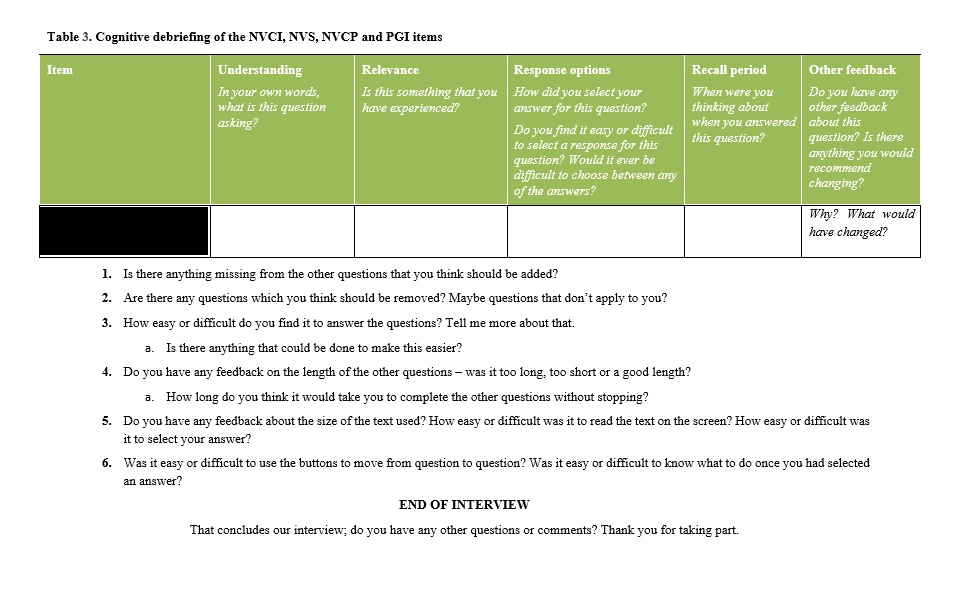

Supplement: Supplementary file 2 — Additional file 2. Example HCP interview guide questions. [file 41687_2021_379_MOESM2_ESM.docx]
